# An encoding generative modeling approach to dimension reduction and covariate adjustment in causal inference with observational studies

**DOI:** 10.1073/pnas.2322376121

**Published:** 2024-05-29

**Authors:** Qiao Liu, Zhongren Chen, Wing Hung Wong

**Affiliations:** ^a^Department of Statistics, Stanford University, Stanford, CA 94305; ^b^Bio-X Program, Stanford University, Stanford, CA 94305; ^c^Department of Statistics and Data Science, Yale University, New Haven, CT 06520; ^d^Department of Biomedical Data Science, Stanford University, Stanford, CA 94305

**Keywords:** average dose–response function, average treatment effect, potential outcome, dimension reduction, deep generative models

## Abstract

In this article, we develop CausalEGM, a deep learning framework for nonlinear dimension reduction and generative modeling of the dependency among covariate features affecting treatment and response. CausalEGM can be used for estimating causal effects in both binary and continuous treatment settings. By learning a bidirectional transformation between the high-dimensional covariate space and a low-dimensional latent space and then modeling the dependencies of different subsets of the latent variables on the treatment and response, CausalEGM can extract the latent covariate features that affect both treatment and response. By conditioning on these features, one can mitigate the confounding effect of the high dimensional covariate on the estimation of the causal relation between treatment and response. In a series of experiments, the proposed method is shown to achieve superior performance over existing methods in both binary and continuous treatment settings. The improvement is substantial when the sample size is large and the covariate is of high dimension. Finally, we established excess risk bounds and consistency results for our method, and discuss how our approach is related to and improves upon other dimension reduction approaches in causal inference.

## Significance

Causal inference has been increasingly essential in modern observational studies with rich covariate information. However, it is often challenging to estimate the causal effect with high-dimensional covariates. Here, we introduce an approach by encoding generative modeling (EGM) for handling high-dimensional covariates by a dependency-aware dimension reduction strategy where the key idea is to identify a latent covariate feature set (e.g., latent confounders) that affects both treatment and outcome. EGM provides a flexible and powerful framework for us to develop deep learning-based estimates for the structural equation modeling that describes the causal relations among variables. Comprehensive numerical experiments suggest that the proposed method is effective and scalable in estimating the causal effect of one variable on another under various settings.

Given data in an observational study, a central problem in causal inference is to estimate the effect of one variable (e.g., treatment) on another variable (e.g., outcome) in the presence of a covariate vector that represents all other variables observed in the study (1–3). Under the well-known “unconfoundedness” condition (4, 5), which assumes that there are no hidden confounding variables beyond the observed covariate vector, valid estimates of the desired effect of treatment on outcome can be obtained by alternative approaches, including matching, weighting, stratification, and regression-based methods (6). Covariate adjustment plays an important role in these methods (7, 8). A common goal in covariate adjustment is to obtain the average dose–response function, which often involves the estimation of the expectation of the outcome conditional on the treatment and the covariate. When the covariate is of high dimension, as is often the case in modern applications (9–11), covariate adjustment becomes difficult because of the “curse of dimensionality” (12).

Various types of dimension reduction approaches have been proposed to alleviate this difficulty. For example, a popular approach is to do adjustment or matching based on the propensity score (4, 13, 14), which is a one-dimensional feature (i.e., a scalar function) of the covariates that captures how the covariates affect the treatment. Of course, the propensity score function must first be learned from the observed data on the treatment and the covariates, which is usually done by logistic regression or other advanced machine learning methods (15). Another type of dimension reduction method is “sufficient dimension reduction” (16, 17) (SDR), which assumes that the treatment assignment is conditionally independent of potential outcomes given the low-dimensional projection of the covariates (18, 19). However, SDR-based causal inference approaches consider only linear dimension reduction which limits the applicability. Furthermore, the dimension reduction is performed separately for each treatment value, which makes it difficult to extend the method to the case when the range of the treatment variable is of high cardinality or is continuous.

The present work develops a covariate adjustment method based on an encoding generative modeling (EGM) approach, called CausalEGM, which simultaneously learns to i) embed the high-dimensional covariates into a low-dimensional latent space where the distribution of the embeddings (latent covariate features) is prespecified. ii) build generative models for treatment given latent features and for outcome given treatment and latent features. The key idea of this method is to partition the latent feature vector into different independent components that play different roles in the above two generative models. This partitioning then allows us to identify a minimal latent covariate feature subvector that affects both treatment and outcome. After presenting the model, we will explain how this can be viewed as an approach to constructing good latent covariate features for covariate adjustment. We will also discuss the difference between our method and alternative dimension reduction methods. In particular, the results of our study show that, by adding the generator function to reconstruct the covariate, our approach achieves better performance than approaches that only focus on extracting low-dimensional covariate features to use in the prediction of treatment and outcome.

In implementing our method, we use multilayer neural networks to represent the encoder and generator functions in the model, which allows us to leverage advances in generative AI in the learning of our model. There has been increasing attention on the use of machine learning in causal inference (20–22). However, most of these methods either learn a predictor of the outcome conditional on the joint state of treatment and covariate or build separate outcome prediction models for each fixed value of the treatment. As such, they are different in nature from the dimension reduction approach which tries to learn a low-dimensional covariate feature to replace the original covariate in the adjustment. We present numerical experiments to demonstrate that our dimension reduction approach will lead to better estimates of the causal effect. Finally, we study the theoretical properties of our approach and establish excess risk bounds and consistency results for our estimates.

## Methods

### Problem Setup.

We are interested in the causal effect of a variable X on another variable Y in an observational study based on i.i.d. observations of $\left\{\left(X_{i},Y_{i},V_{i}\right)|i=1,...,n\right\}$. X is usually called the treatment (or exposure) variable, and Y is called the response (or outcome) variable. $V\in R^{p}$ represents the covariates in a p-dimensional space. Y is real-valued in the Y outcome space and X∈X, where the support X is either a finite set or a bounded interval in R.

To investigate causal effects, we aim to determine how the potential outcome will respond to the change of treatment, which is given by the function Y(·):X→R. We are particularly interested in estimating the population average defined as

[1] $$\begin{array}{c} \mu (x)=E[Y(x)], \end{array}$$

which is known as the average dose–response function (ADRF). Note that we only observe the potential outcome indexed by the treatment variable. The random variable Y(x) is not directly observable, and its expectation μ(x) is generally not identifiable from the joint distribution of the observed (X,Y,V). Additional assumptions are needed for the identification of μ(x).

We first assume X and Y are related by the following two unknown equations:

[2] $$\begin{array}{c} \left\{\begin{array}{cc} X= & h_{0}\left(Z_{x},U_{1}\right),\\ Y= & f_{0}\left(X,Z_{y},U_{2}\right), \end{array}\right. \end{array}$$

where $U=(U_{1},U_{2})$ represents the set of all other unobserved variables that may affect X and Y, which include disturbances. $Z_{x}=t_{x}\left(V\right)$ and $Z_{y}=t_{y}\left(V\right)$ are two low-dimensional feature sets of V and $t_{x}\left(\cdot \right)$, $t_{y}\left(\cdot \right)$ are the corresponding transformation functions. We denote $Z_{0}=Z_{x}\cap Z_{y}$ as the intersection of the two feature sets. We will assume a modified version of the “unconfoundedness” condition.

Assumption 1. ***(Unconfoundedness)*** *Conditional on the low-dimensional feature set* $Z_{0}$*, the potential outcomes* Y(x) *is independent of treatment variable* X,

[3] $$\begin{array}{c} X⫫Y\left(x\right)|Z_{0}. \end{array}$$

Since Y(x) is a function of $Z_{y}$ and $U_{2}$, the above assumption is also equivalent to $X⫫\left\{Z_{y},U_{2}\right\}|Z_{0}$. Note that under the conventional unconfoundedness assumption, one must condition on the high-dimensional covariates V. Under our *Assumption* 1, it is sufficient to condition on a low-dimensional feature set of the covariates. Once $Z_{0}$ is given, there should be no unobserved confounding variables that drive correlated changes between the treatment and the outcome variables.

Under *Assumption* 1, it is shown that the ADRF is identifiable through the following equation (*SI Appendix*, section A),

[4] $$\begin{array}{c} \mu \left(x\right)=\int E\left[Y|X=x,Z_{0}=z_{0}\right]p_{Z_{0}}\left(z_{0}\right)dz_{0}. \end{array}$$

The equation **4** shows that we can replace the original covariate V by a low-dimensional covariate feature $Z_{0}\left(V\right)$, the causal inference problem is transformed into the problem of learning a low-dimensional representation of V from the observational data. To learn this transformation function, we propose a EGM framework, that allows simultaneous learning of an encoder for the high-dimensional V and a generative model for (X,Y,V). By imposing a suitable constraint on the generative model, one can ensure that certain subsets of the features computed by the encoder can be used as the low-dimensional feature $Z_{0}$ in the above condition. In the next subsection, we will illustrate how to use the neural networks to learn the low-dimensional features $Z_{0}$ and estimate the μ(x) in Eq. **4**.

### EGM.

**Fig. 1.**
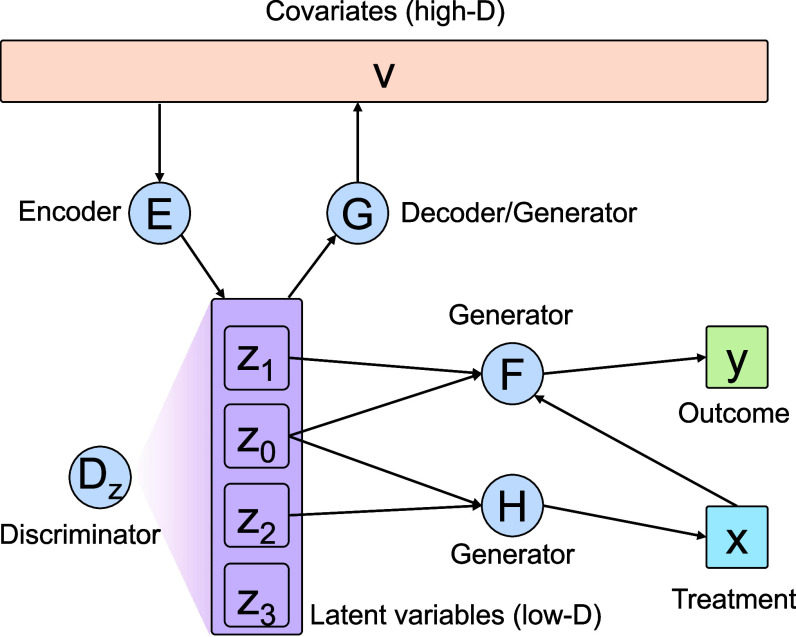
The overview of CausalEGM model. Variables are in rectangles. Functions are in circles, with incoming arrows indicating inputs to the function and outgoing arrows indicating outputs of the function. Each function is modeled by a neural network. CausalEGM takes triplets of (X,Y,V) as input. E and G networks form a bidirectional transformation between the high-dimensional covariates space and a low-dimensional latent space. $D_{z}$ network is used to constrain the distribution of latent features. F and H are used for generating/reconstructing the outcome and treatment variables, respectively. As G, F, and H networks take latent variables as input(s) with a desired distribution, they are also known as generators.

Our model is described in Fig. 1. To handle the high dimension of V, we embed V into a low-dimensional latent space using an encoder function Z=E(V) and a generator/decoder function V=G(Z) to map Z back to the original space. Note that dimension reduction with controllable latent features has been successfully applied in our previous works, including density estimation (23) and clustering (24).

In a standard autoencoder, G and E functions are learned by minimizing the reconstruction error between G(E(V)) and V over the observed sample of V. Here, E(V) represents the low-dimensional latent features. However, it is important to consider the complex dependencies of covariates on treatment and outcome and the generation process of treatment and outcome. The proposed EGM framework simultaneously enables a dependency-aware dimension reduction and modeling the generation process of treatment and outcome. It is natural to suppose there are independent covariate feature sets A, B, and C with different roles. A is involved in the generation of both outcome and treatment, B is involved only in outcome generation, and C is involved only in the treatment generation. We can usually find invertible transformations of each to a separate standard multivariate normal vector, denoted as $Z_{0}$, $Z_{1}$, and $Z_{2}$ when V has a continuous distribution. We also demonstrated that EGM framework can handle the discrete covariates distribution well. In fact, any one-to-one transformation of A, B, and C can be used for covariates and the form of the generator functions of treatment and outcome will change depending on which transformation functions are used. However, all of them will lead to the same covariate adjustment because the conditional expectation of the outcome will remain the same.

To achieve the above goal, we impose a “distribution-matching” objective in addition to the reconstruction error. Specifically, we desire that the distribution of Z=E(V) should match a prespecified distribution, which is set to be a standard normal distribution. By the encoding process, the high-dimensional covariates with unknown distribution will be mapped to a low-dimensional latent space with a desired distribution. More importantly, we partition the latent feature vector into different subvectors that play different roles in the generative models for treatment and outcome. This partitioning enables us to identify a minimal covariate feature (e.g., $Z_{0}$) that affects both treatment and outcome. In addition to $Z_{0}$, $Z_{1}$, and $Z_{2}$, we find that adding a flexible $Z_{3}$ that affects neither treatment nor outcome is useful to improve the learning of the confounding features and the generative models for treatment and outcome. We use deep neural networks to represent the functions E(·) and G(·). We utilized generative adversarial networks (GANs) (25) for distribution match where an adversarial loss (i.e., maximizing the discrimination power between the generated and observed data) is introduced.

Note that the learning of E(·) and G(·) should not be based on V alone. Rather, they must be coupled with the learning of generative models for X and Y, which are the variables of interest in causal inference. To do this, we assume that the Z=E(V) can be partitioned into different subvectors that have different roles in the generators for X and Y. Specifically, $Z=(Z_{0},Z_{1},Z_{2},Z_{3})$, $Y=F\left(X,Z_{0},Z_{1}\right)+\epsilon_{1}$ and $X=H\left(Z_{0},Z_{2}\right)+\epsilon_{2}$. For clarity, the additive independent noises $\epsilon_{1}$ and $\epsilon_{2}$ are omitted in Fig. 1. Conceptually, $Z_{0}$ represents the latent covariate features that affect both treatment and outcome (e.g., confounding features), $Z_{1}$ represents the latent features that affect only the outcome, $Z_{2}$ represents the latent features that affect only the treatment, and $Z_{3}$ represents the remaining latent features that are also important for the representation of V. By partitioning the latent features Z into four different components, the encoder function decouples the complex dependencies of covariants on treatment and outcome variables in the low-dimensional latent space.

The training details are given in the next subsection for how to jointly learn (F,H,E,G) in an end-to-end fashion given the observational data. Assuming that the four functions (F,H,E,G) are learned, the latent features Z then can be easily extracted through the encoder function E(·). To estimate the average dose–response function μ(x), the simplest way is to fit a nonparametric regression to estimate the conditional expectation in Eq. **2** and then calculate the empirical expectation given the observational data. In practice, we can also use

[5] $$\begin{array}{c} \hat{\mu }\left(x\right)=\frac{1}{n}\sum_{i=1}^{n}F\left(X=x,Z_{0}=z_{0}^{\left(i\right)},Z_{1}=z_{1}^{\left(i\right)}\right), \end{array}$$

to estimate the ADRF where n is the sample size. In binary treatment settings, the counterfactual (CF) outcome for the $i^{\mathrm{th}}$ unit is estimated as

[6] $$\begin{array}{c} y_{\mathrm{CF}}^{\left(i\right)}=F\left(X=1-x^{\left(i\right)},Z_{0}=z_{0}^{\left(i\right)},Z_{1}=z_{1}^{\left(i\right)}\right). \end{array}$$

### Model Training.

The CausalEGM model consists of a bidirectional transformation module (E, G) and two additional networks (F, H) for reconstructing/generating outcome and treatment, respectively (Fig. 1). The bidirectional module is a combination of an autoencoder and a GAN model. In addition to the reconstruction error between G(E(V)) and V in the covariates space commonly required by the autoencoder, the encoder network E aims to transform the covariates into latent features, whose distribution matches the standard multivariate normal distribution. A discriminator $D_{z}$ network tries to distinguish data sampled from the multivariate normal distribution (positives) from data generated by the E network (negatives) where (E, $D_{z}$) forms a GAN model. Similarly, it is optional to minimize reconstruction error between E(G(Z)) and Z in the latent space and introduce another discriminator $D_{v}$ in the covariate space to form a GAN model (G, $D_{v}$) to match the empirical distribution of the covariates V and the reconstructed data by G. We use the Wasserstein GAN with gradient penalty (26) as the default architecture to improve the generation power and training stability.

Thus, the loss functions of the adversarial training for distribution matching in latent space are represented as

[7] $$\begin{array}{c} \left\{\begin{array}{cc} L_{E}= & -E_{v∼\hat{p}\left(v\right)}\left[D_{z,-1}\left(E\left(v\right)\right)\right],\\ L_{D_{z}}= & -E_{z∼p(z)}\left[D_{z,-1}\left(z\right)\right]+E_{v∼\hat{p}\left(v\right)}\left[D_{z,-1}\left(E\left(v\right)\right)\right]\\ & +\lambda E_{z∼\bar{p}\left(z\right)}\left[{\left(\nabla D_{z,-1}\left(z\right)-1\right)}^{2}\right], \end{array}\right. \end{array}$$

where p(z) and $\hat{p}\left(\cdot \right)$ denote the standard normal distribution and the empirical distribution, respectively. To make the output of discriminator $D_{z}$ differentiable, we use $D_{z,-1}\left(\cdot \right)$ to denote the output before binarization, which is achieved by a sigmoid function. $\bar{p}\left(z\right)$ denotes the uniform sampling from the straight lines between the two points sampled from the standard normal distribution p(z) and the empirical distribution of latent features $\hat{p}\left(z\right)$. The network E and $D_{z}$ are competing with each other during the adversarial training until reaching a Nash equilibrium. λ is the gradient penalty coefficient, which is set to 10 in all experiments.

In addition to adversarial training for distribution match, the reconstruction losses for (X,Y,V) are denoted as

[8] $$\begin{array}{c} \left\{\begin{array}{cc} L_{\mathrm{rec}}^{x}= & E_{x∼\hat{p}\left(x\right),z_{0}∼{\hat{P}}_{e_{0}\left(V\right)},z_{2}∼{\hat{P}}_{e_{2}\left(V\right)}}\left[{\left(x-F\left(z_{0},z_{2}\right)\right)}^{2}\right],\\ L_{\mathrm{rec}}^{y}= & E_{y∼\hat{p}\left(y\right),z_{0}∼{\hat{P}}_{e_{0}\left(V\right)},z_{1}∼{\hat{P}}_{e_{1}\left(V\right)}}\left[{\left(y-H\left(z_{0},z_{1},x\right)\right)}^{2}\right],\\ L_{\mathrm{rec}}^{v}= & E_{v∼\hat{p}\left(v\right)}[||v-{G\left(E\left(v\right)\right)||}_{2}^{2}]. \end{array}\right. \end{array}$$

where ${\hat{P}}_{e_{k}\left(V\right)}$ (k=0,1,2,3) is the empirical distribution of the $k^{\mathrm{th}}$ component of the encoder e(V) and $\left|\right|\cdot {\left|\right|}_{2}^{2}$ denotes the squared $l^{2}$-norm. The total loss for CausalEGM can be summarized into two parts $L_{\mathrm{FHEG}}=L_{E}+L_{\mathrm{rec}}^{x}+L_{\mathrm{rec}}^{y}+L_{\mathrm{rec}}^{v}$ and $L_{D_{z}}$, which correspond to the major four networks (F,H,E,G) and the auxiliary discriminator network $D_{z}$, respectively. To train the CausalEGM model in an end-to-end fashion, we iteratively update the parameters (weights) in one of (F,H,E,G) or $D_{z}$ given the value of the other. Each iteration contains the following two steps. In the first step, a minibatch of data is randomly sampled and the parameters of discriminator $D_{z}$ are updated by minimizing $L(D_{z})$ while fixing the parameters in (F,H,E,G). In the second step, a minibatch of data is randomly sampled and the parameters of (F,H,E,G) are updated by minimizing $L_{\mathrm{FHEG}}$ while fixing the parameters in $D_{z}$.

### Model Architecture.

For all numerical examples below, we use fully connected layers for all networks. Specifically, the (F,H,E,G) networks contain five fully connected layers, and each layer has 64 hidden nodes. The $D_{z}$ network contains three fully connected layers with 64, 32, and 8 hidden nodes, respectively. The leaky-ReLu activation function is deployed as a nonlinear transformation in each hidden layer. We use Sigmoid as the activation function in the last layer of H network when the treatment is binary. For continuous treatments, we do not use any nonlinear activation function in the last layer of H network. Batch normalization (27) is applied in discriminator networks. Adam optimizer (28) with initial learning rate as $2\;\times \;10^{-4}$ is used. The model parameters were updated in a minibatch manner with batch size 32. The default number of training iterations is 30,000.

## Theoretical Analysis

### GAN background.

Let P and Q be two probability measures and A be a class of measurable subsets of the space U. Then define $d\left(P,Q;A\right):=\underset{A\in A}{\sup}\left|P\left(A\right)-Q\left(A\right)\right|$. Note that the function d(·) defines a pseudodistance function between two probability measures. For example, if we let B be the Borel sets, d(P,Q;B) would become the variation distance between P and Q. Suppose P and Q have densities p and q, we then have $d\left(P,Q;B\right)=\frac{1}{2}||p-{q||}_{L_{1}}$.

Let $A_{M}:=\left\{A\in A:\exists D\in D_{M}\;\text{s.t.}\forall u\in A,D\left(u\right)=1\right\}$, where D:U→{0,1} indicates a discriminator (classifier) and $D_{M}$ is the set of discriminators constructed by deep neural networks with complexity parameter M (M can represent the number of layers, numbers of hidden nodes, etc). In general, M increases when sample size n goes large. If $Q_{G}$ is the probability measure induced by the generative model G(Z), the adversarial training is then equivalent to minimizing the pseudodistance between the induced distribution and the empirical distribution

[9] $$\begin{array}{c} \underset{G}{\inf}\underset{A\in A_{M}}{\sup}\left|Q_{G}\left(A\right)-\hat{P}\left(A\right)\right|=\;\underset{G}{\inf}d\left(Q_{G},\hat{P};A_{M}\right), \end{array}$$

where $\hat{P}\left(u\right)$ is the empirical distribution given by $\frac{1}{n}\sum_{i=1}^{n}\delta_{u_{i}}\left(u\right)$, where $\left\{u_{i}|i=1,...,n\right\}$ is the observed data and $\delta_{u}$ is the Dirac measure.

### Empirical Risk Minimization.

The empirical risk terms are represented as

[10] $$\begin{array}{c} \left\{\begin{array}{cc} L_{1}= & E_{n}\left[{\left(Y-f\left(X,e_{0}\left(V\right),e_{1}\left(V\right)\right)\right)}^{2}\right],\\ L_{2}= & E_{n}\left[{\left(X-h\left(e_{0}\left(V\right),e_{2}\left(V\right)\right)\right)}^{2}\right],\\ L_{3}= & \underset{A\in A_{M}}{\sup}\left|P\left(A;Z^{0}\right)-\hat{P}\left(A;e\left(V\right)\right)\right|=d\left({\hat{P}}_{e(V)},P_{Z^{0}};A_{M}\right),\\ L_{4}= & E_{n}[||V-{g\left(e\left(V\right)\right)||}_{2}^{2}], \end{array}\right. \end{array}$$

where $E_{n}$ denotes the empirical expectation based on the observed data with sample size n. $P_{Z^{0}}$ is the probability measures of $Z^{0}∼N\left(0,I\right)$ and ${\hat{P}}_{e(V)}$ is the empirical distribution of e(V). The empirical risk is denoted as $R_{\mathrm{emp}}\left(f,h,e,g\right)=L_{1}+L_{2}+L_{3}+L_{4}$.

The corresponding true risk is $R^{0}\left(f,h,e,g\right)=R_{1}^{0}+R_{2}^{0}+R_{3}^{0}+R_{4}^{0}$, where

[11] $$\begin{array}{c} \left\{\begin{array}{cc} R_{1}^{0}= & E_{0}\left[{\left(Y-f\left(X,Z_{0},Z_{1}\right)\right)}^{2}\right],\\ R_{2}^{0}= & E_{0}\left[{\left(X-h\left(Z_{0},Z_{2}\right)\right)}^{2}\right],\\ R_{3}^{0}= & d(P_{e(V)},P_{Z^{0}};A_{M}),\\ R_{4}^{0}= & E_{0}[||V-{g\left(e\left(V\right)\right)||}_{2}^{2}]. \end{array}\right. \end{array}$$

where $E_{0}$ stands for the population expectation with respect to the underlying distribution of the random variables and $P_{e(V)}$ is the probability measure induced by e(V). We denote $F_{M}$ and $D_{M}$ as the function classes of the neural network generator/encoder and discriminator with complexity M, respectively. By minimizing empirical risk (MER), we obtain the empirical solution as

[12] $$\begin{array}{c} {\hat{f}}_{M,n},{\hat{h}}_{M,n},{\hat{e}}_{M,n},{\hat{g}}_{M,n}=\underset{f,h,e,g\in F_{M}}{\arg\;\min}R_{emp}(f,h,e,g). \end{array}$$

### Rademacher Complexity.

We use Rademacher complexity to measure the richness of a function class w.r.t. a probability distribution. The empirical Rademacher complexity term is defined as

[13] $$\begin{array}{c} \begin{array}{c} R_{n}\left(F\right):=E_{\sigma }\left[\underset{F\in F}{\sup}\frac{1}{n}\sum_{i=1}^{n}\sigma_{i}F\left(X_{i}\right)\right], \end{array} \end{array}$$

where $\sigma_{i}$ is i.i.d. drawn from the Rademacher distribution with $P\left(\sigma_{i}=1\right)=P\left(\sigma_{i}=-1\right)=\frac{1}{2}$.

### Excess Risk.

We can now define the excess risk within the function class $F_{M}$ as

[14] $$\begin{array}{c} R^{0}\left({\hat{f}}_{M,n},{\hat{h}}_{M,n},{\hat{e}}_{M,n},{\hat{g}}_{M,n}\right)-\underset{f,h,e,g\in F_{M}}{\inf}R^{0}\left(f,h,e,g\right). \end{array}$$

We then characterize the convergence rate of the excess risk in terms of the Rademacher complexity of the function classes from the empirical risk terms.

Theorem 1. *(**Bound of Excess Risk**)* *Under our problem setup with bounded input domain,* $F_{M}$ *and* $D_{M}$ *are the classes of neural network encoder/generator and discriminator with complexity M used in our model, respectively. We also add the composition functions of encoder and generator, such as * g ○ e, to $F_{M}$*. Let* $L_{\lambda }$ *be the* λ*-Lipschitz squared loss function used in the empirical risk terms. Assume that* $F_{M}$ *and* $D_{M}$ *are uniformly equi-continuous and* b*-uniformly bounded, respectively.* $R_{n}\left(\cdot \right)$ *denotes the empirical Rademacher complexity. For any* δ>0*, we have*

[15] $$\begin{array}{c} R^{0}({\hat{f}}_{M,n},{\hat{h}}_{M,n},{\hat{e}}_{M,n},{\hat{g}}_{M,n})-\underset{f,h,e,g\in F_{M}}{\inf}R^{0}(f,h,e,g)\\ \;\;\;\;\;\leq (8+4p)R_{n}(L_{\lambda }○F_{M})+4R_{n}(D_{M})+\delta \end{array}$$

with probability at least $1-4e^{-\frac{n\delta^{2}}{32b^{2}{\left(3+p\right)}^{2}}}$,

where the operator ○ denotes the function composition and p is the number of covariates. The detailed proof is given in (*SI Appendix*, section B). Theorem 1 gives a high probability bound of the excess risk, which involves the calculation of Rademacher complexity. The Rademacher complexity can be further upper bounded in terms of the complexity of the class, such as covering number or Vapnik–Chervonenkis dimension. See refs. 29 and 30 for details from the viewpoint of the empirical process. Bounding the Rademacher complexity of deep neural networks has also been widely explored. For example, Truong (31) provided an order $O(1/\sqrt{n})$ for bounding the Rademacher complexity of feedforward neural network with finite depth and width (see its theorem 5). Li et al. (32) further showed that given a λ-Lipschitz continuous loss function $L_{\lambda }$, then $R_{n}\left(L_{\lambda }○F_{M}\right)$ has the order of $\tilde{O}\left(\lambda \sqrt{\mathrm{DWr}}/\sqrt{n}\right)$, where $\tilde{O}\left(\cdot \right)$ represents the rate by ignoring logarithmic factors and D,W, and r represent the depth, width, and rank of weight matrices in the neural network, respectively (see its theorem 1). Since generators, encoder, and discriminator in CausalEGM are all fully connected neural networks, the excess risk from the left-hand side of Eq. **15** converges to zero almost surely as long as the Rademacher complexity terms are in the order of o(1).

### Consistency.

Under an assumption on the encoder–decoder networks related to dimension reduction, we can show the consistency of our empirical solution in formula **12**.

Assumption 2. *There exists* ${\tilde{e}}_{3}$, $\tilde{g}$*, and* δ>0 *s.t.*

[16] $$\begin{array}{c} \left(e_{0}^{0},e_{1}^{0},e_{2}^{0},{\tilde{e}}_{3}\right)\overset{D}{=}Z^{0}. \end{array}$$

*For any function* e *and* g*, we have*

[17] $$\begin{array}{c} E_{0}[||V-\tilde{g}\left(\left(e_{0}^{0},e_{1}^{0},e_{2}^{0},{\tilde{e}}_{3}\right)\left(V\right)\right){\left|\right|}_{2}^{2}]\leq E_{0}[||V-g\left(\left(e\right)\left(V\right)\right){\left|\right|}_{2}^{2}]+\delta . \end{array}$$

The left-hand side of the inequality in Eq. **17** denotes the reconstruction error with the “distribution match” constraint while the first term on the right-hand side of the inequality in Eq. **17** denotes the reconstruction error in a dimension reduction framework without any constrain. The constant delta is the “price” for adding the distribution match constraint in the latent space. This assumption is expected to hold with a small delta when the distribution of V satisfies a certain “dimension reduction” property. We provide a concrete example in *SI Appendix*, section C to demonstrate the rationale of this assumption.

Based on the above assumption, we can derive the consistency theorem as follows.

Theorem 2. *(**Consistency**)* *Under the same setting as Theorem 1, suppose* $\cup_{M}F_{M}$ *and* $\cup_{M}D_{M}$ *are uniformly equi-continuous and* b*-uniformly bounded, respectively. Let* $\left(f^{*},h^{*},e^{*},g^{*}\right)$ *be any limit point of MER solution* ${\left(\hat{f},\hat{h},\hat{e},\hat{g}\right)}_{n}$ *when* n→∞. *If Assumption 1, 2 hold and the Rademacher complexity terms in Theorem 1 go to zero as the sample size* n *and the model complexity* M *increase, we have*

[18] $$\begin{array}{c} \begin{array}{c} E_{0}\left[{\left(\left(f^{0}-f^{*}\right)\left(X,Z_{0},Z_{1}\right)\right)}^{2}\right]\;+\&E_{0}\left[{\left(\left(h^{0}-h^{*}\right)\left(Z_{0},Z_{2}\right)\right)}^{2}\right]\\ +\&d(P_{Z^{0}},P_{e^{*}\left(V\right)};A_{M})\leq 2\delta . \end{array} \end{array}$$

*Theorem* 2 suggests that if V can be encoded effectively s.t. the *Assumption* 2 is satisfied with δ≈0, we would have approximately

[19] $$\begin{array}{c} f^{*}\approx f^{0},h^{*}\approx h^{0},e^{*}\left(V\right)\overset{D}{\approx }Z^{0}. \end{array}$$

This holds for any limit points of $\left\{{\left(\hat{f},\hat{h},\hat{e},\hat{g}\right)}_{n}\right\}$. The detailed proof is given in (*SI Appendix*, section D).

## Results

We performed a series of experiments to evaluate the performance of CausalEGM against state-of-the-art methods under different settings. In the continuous treatment setting, we test the performance of CausalEGM in learning the average dose–response function (ADRF). In the binary treatment setting, we aim to verify the ability of CausalEGM to estimate both the average treatment effect (ATE) and the individual treatment estimation (ITE).

### Datasets.

For the continuous treatment setting, four different datasets from previous publications (14, 33, 34) were used, including three simulated datasets and one semisynthetic dataset. The semisynthetic dataset was collected from 71,345 twins where the weight is the continuous treatment variable and we simulate the risk of death (outcome) under a model in which higher weight leads to a lower death rate in general.

For the binary treatment setting, we used the datasets from the 2018 Atlantic Causal Inference Conference (ACIC) competition. This dataset utilizes the linked births and infant deaths database (LBIDD) based on real-world medical measurements. The LBIDD data are semisynthetic, where 117 measured covariates are given, and the treatment and outcome are simulated based on different data-generating processes. We selected nine datasets by using the most complicated generation process (e.g., the highest degree of generation function) with sample sizes ranging from 1,000 to 50,000. The details of all datasets used in this paper were provided at (*SI Appendix*, section E).

### Model Evaluation.

In the continuous treatment setting, we aim to evaluate whether the estimated dose–response function μ(x) can well approximate the true dose–response function. Two commonly used metrics, including RMSE and mean absolute percentage error (MAPE) are used where

[20] $$\begin{array}{c} \left\{\begin{array}{cc} RMSE= & \sqrt{\frac{1}{n}\sum_{i=1}^{n}{\left(\mu \left(x_{i}\right)-\hat{\mu }\left(x_{i}\right)\right)}^{2}},\\ MAPE= & \frac{1}{n}\sum_{i=1}^{n}\left|\frac{\mu \left(x_{i}\right)-\hat{\mu }\left(x_{i}\right)}{\mu (x_{i})}\right|. \end{array}\right. \end{array}$$

In the binary treatment setting, we use absolute error of average treatment effect ($\epsilon_{\mathrm{ATE}}$) and mean squared error of precision in estimation of heterogeneous effect ($\epsilon_{\mathrm{PEHE}}$) for evaluating the performance where

[21] $$\begin{array}{c} \left\{\begin{array}{cc} \epsilon_{\mathrm{ATE}}= & |\frac{1}{n}\sum_{i=1}^{n}\left({\hat{Y}}_{i}\left(1\right)-{\hat{Y}}_{i}\left(0\right)\right)-\frac{1}{n}\sum_{i=1}^{n}\left(Y_{i}\left(1\right)-Y_{i}\left(0\right)\right)|,\\ \epsilon_{\mathrm{PEHE}}= & \frac{1}{n}\sum_{i=1}^{n}{\left({\hat{Y}}_{i}\left(1\right)-{\hat{Y}}_{i}\left(0\right)-\left(Y_{i}\left(1\right)-Y_{i}\left(0\right)\right)\right)}^{2}. \end{array}\right. \end{array}$$

Note that ${\hat{Y}}_{i}\left(\cdot \right)$ denotes the predicted/imputed value of potential outcome.

### Baselines.

For the continuous treatment setting, three different baselines, including ordinary least squares regression (OLS), regression prediction estimator (REG) (35, 36), and double debiased machine learning estimators (34) were used. For the binary treatment setting, we compared CausalEGM to neural network-based methods [CFR (37), Dragonnet (38), CEVAE (39) and GANITE (40)], tree-based methods CausalForest (41), and sufficient dimension reduction based method SDRcausal (42). A detailed introduction of these competing methods was provided in *SI Appendix*, section F.

### Continuous Treatment Experiments.

**Fig. 2.**
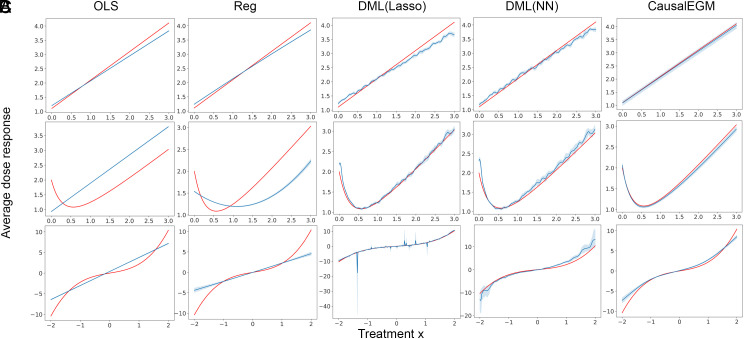
The performance of CausalEGM and baseline methods (OLS, Reg, DML with Lasso or neural network) under continuous treatment settings across three benchmark datasets. (*A*) Hiranos and Imbens dataset. (*B*) Sun et al. dataset. (*C*) Colangelo and Lee dataset. The red curves are the ground truth while the blue curves are the estimated average dose–response with 95% CI based on 10 independent simulations.

We first evaluate the performance of the CausalEGM model in the continuous setting where the treatment x∈X and X is a bounded interval in R. We set the sample size and number of covariates to be 20,000 and 200 in the simulated datasets, respectively. The latent dimensions (dimension of $z_{i}$ where i=0,1,2,3) of the four datasets are set to be (1,1,1,7), (2,2,2,4), (5,5,5,5), and (1,1,1,7), respectively. It is shown that CausalEGM demonstrates superior results over the existing methods, including two linear regression-based methods OLS and REG, and a kernel-based machine learning approach with two different machine learning algorithms (lasso and neural network). We first evaluate whether the dose–response function can be well estimated by different competing methods (Fig. 2). It is observed that OLS and Reg result in relatively large estimation errors. The dose–response curves estimated by the DML methods have spikes and fluctuations. In contrast, the curves estimated by CausalEGM are smooth and the estimation errors are small.

In terms of the quantitative measurements, CausalEGM achieves the lowest RMSE and MAPE in all three simulated datasets compared to baseline methods (Table 1). We also note that DML method performs much better than linear regression-based methods (OLS and REG) in Hiranos and Imbens and Twins datasets while performing less well in the other two. CausalEGM reduces the RMSE, MAPE by 24.2% to 63.4%, 6.9% to 55.2% compared to the best baseline method across different datasets, respectively. The results of both simulated data and real data illustrate that CausalEGM offers significant improvement for estimating the causal effect in continuous settings.

**Table 1.** Result on continuous treatment setting

Dataset	Method	RMSE	MAPE
Imbens et al.	OLS	0.680±0.0	0.367±0.0
	REG	0.525±0.0	0.214±0.0
	DML(lasso)	0.090±0.0	0.037±0.0
	DML(nn)	0.133±0.022	0.052±0.011
	CausalEGM	0.041±0.014	0.019±0.006
Sun et al.	OLS	0.140±0.0	0.041±0.0
	REG	0.117±0.0	0.039±0.0
	DML(lasso)	0.163±0.0	0.050±0.0
	DML(nn)	0.0970±0.0190	0.0346±0.006
	CausalEGM	0.0738±0.0399	0.0345±0.0170
Lee et al.	OLS	1.3±0.0	1.2±0.0
	REG	1.5±0.0	0.565±0.0
	DML(lasso)	0.487±0.0	0.168±0.0
	DML(nn)	1.3±0.581	0.494±0.181
	CausalEGM	0.125±0.040	0.119±0.080
Twins	OLS	0.109±0.0	0.260±0.0
	REG	11±0.0	64±0.0
	DML(lasso)	0.075±0.0	0.165±0.0
	DML(nn)	0.059±0.002	0.158±0.006
	CausalEGM	0.0339±0.020	0.090±0.053

Each method was run for 10 times and the SD was also shown. The best performance is marked in bold.

### Binary Treatment Experiments.

In the binary treatment settings where the treatment x∈{0,1}, we aim to evaluate whether CausalEGM could estimate an accurate treatment effect. The latent dimensions were set to be (3,6,3,6). CausalEGM was benchmarked against a number of state-of-the-art methods on the ACIC 2018 benchmark datasets, which provide various simulation settings and sample sizes. We chose three datasets from each of three different sample sizes (1, 10, and 50 K) with the most complicated generation process (e.g., the generation functions are of the highest order/degree). CausalEGM is compared to six baseline methods on each of these datasets. As shown in Table 2, CausalEGM achieves the smallest $\epsilon_{\mathrm{ATE}}$ in six out of nine datasets. CausalEGM performs especially well in datasets with large sample sizes (e.g., 50 K). For example, the $\epsilon_{\mathrm{ATE}}$ is reduced by 16.7% to 98.7% in the three largest datasets compared to the second-best method. For another metric, CausalEGM achieves the smallest $\epsilon_{\mathrm{PEHE}}$ in five out of nine datasets and the second-best performance in the remaining four datasets. To sum up, our model shows superior performance in estimating both average treatment effect and individual treatment effect and is especially powerful when the sample size is large.

**Table 2.** The performance of CausalEGM and comparison methods in ACIC 2018 dataset with various sample sizes

Metric	Dataset	TARNET	CFRNET	CEVAE	GANITE	Dragonnet	CausalForest	CausalEGM
$\epsilon_{\mathrm{ATE}}$	Datasets-1k	0.022±0.015	0.018±0.015	0.035±0.021	0.27±0.08	0.010±0.004	0.021±0.001	0.0097±0.0075
		0.038±0.029	0.041±0.027	0.12±0.10	2.0±0.3	0.012±0.007	0.017±0.003	0.032±0.020
		0.10±0.06	0.095±0.079	0.38±0.27	2.0±1.4	0.16±0.10	0.23±0.02	0.26±0.07
	Datasets-10k	6.4±3.5	12±7	204±58	2.7±1.2	124±11	2.5±1.1	1.3±0.6
		0.056±0.001	0.056±0.001	0.070±0.031	1.2±0.2	0.0097±0.069	0.0057±0.0004	0.0043±0.0025
		0.034±0.023	0.060±0.002	0.018±0.011	0.12±0.09	0.078±0.057	0.013±0.003	0.039±0.016
	Datasets-50k	0.038±0.021	0.085±0.105	0.59±0.31	1.4±0.5	0.89±0.53	0.024±0.003	0.020±0.013
		0.044±0.003	0.045±0.004	0.66±0.59	2.3±0.2	0.027±0.028	0.010±0.001	0.0098±0.0089
		0.30±0.01	0.30±0.01	0.64±0.45	1.9±0.3	0.16±0.08	0.12±0.01	0.0016±0.0010
$\epsilon_{\mathrm{PEHE}}$	Datasets-1k	0.11±0.02	0.00069±0.00075	0.012±0.005	0.14±0.04	0.038±0.003	0.00080±0.00005	0.0069±0.0016
		0.35±0.03	0.29±0.04	0.27±0.04	4.34±1.24	0.34±0.01	0.27±0.01	0.25±0.01
		0.31±0.14	0.28±0.23	7.6±5.3	12±6	1.7±0.4	0.075±0.006	0.20±0.03
	Datasets-10k	433±106	662±288	46200±15500	78.7±26.8	22200±4130	483.72±31.68	7.2±2.6
		0.024±0.005	0.022±0.006	0.091±0.019	2.08±0.45	0.042±0.003	0.015±0.001	0.014±0.001
		0.012±0.005	0.0040±0.0028	0.0034±0.0013	0.14±0.08	0.036±0.015	0.0016±0.0008	0.0028±0.0013
	Datasets-50k	0.88±0.04	0.90±0.08	1.1±0.5	3.4±1.4	1.84±0.83	0.65±0.01	0.55±0.01
		0.031±0.006	0.030±0.011	0.84±0.76	5.454±0.65	0.039±0.007	0.020±0.002	0.022±0.001
		0.22±0.07	0.27±0.05	0.67±0.61	3.8±1.1	0.14±0.06	0.022±0.001	0.0054±0.0013

Each method was run 10 times and the SD are shown. The best performance is marked in bold.

Next, we evaluate whether the EGM framework can learn a better low-dimensional representation compared to sufficient dimension reduction (SDR). Note that all SDR-based methods for causal inference use the linear SDR which is restrictive and may not be able to capture the nonlinear relationship in complex datasets. We made a comprehensive comparison of CausalEGM with SDRcausal under experimental settings either satisfying or violating the SDR assumption. Note that SDRcausal implements several different variants from the original paper (42) and we always choose the best result to report. CausalEGM shows great improvement over SDRcausal in both settings, especially in the nonlinear dataset where a linear SDR failed to work (*SI Appendix*, section G).

### Impact of Discrete Covariates.

CausalEGM has demonstrated superior empirical performance with discrete covariates, such as the ACIC 2018 where all 117 covariates are discrete. One natural question to ask is whether we can always construct standard normal variables in the latent space when some of the covariates are discrete.

From the theoretical perspective, under the condition that there are enough covariates that are independent of both treatment and response, we can construct an approximated standard normal distribution even when the confounders are discrete variables. For example, suppose covariants $V_{1}$, $V_{2}$,...,$V_{p}$ are i.i.d. binary variables with probability $\frac{1}{2}$ on +1 and −1. Assume $V_{1}$ is the true confounder that affects both treatment X and outcome Y while other covariates are not involved in the generative models for X and Y. Then the statistic $W=V_{1}\frac{|\sum_{i=2}^{p}V_{i}|}{\sqrt{p-1}}$ will satisfy the following two conditions. 1) W∼N(0,1) approximately when p is large. 2) W contains all the information in the confounder as $V_{1}$ can be recovered by taking the sign of W. We added this example in *SI Appendix*, section H.

**Fig. 3.**
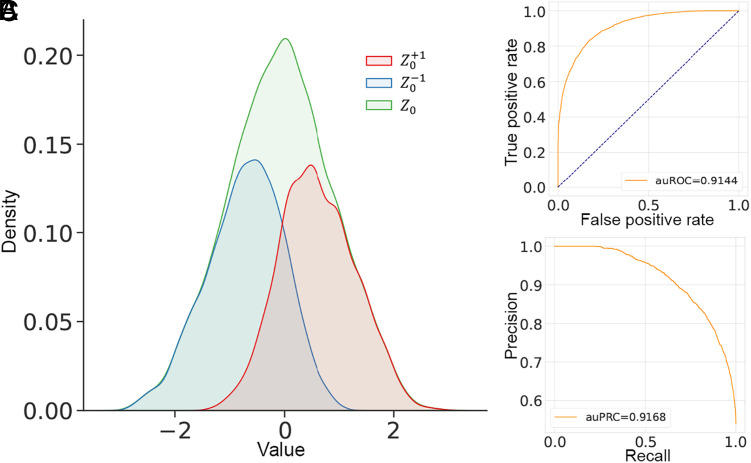
Latent confounding variable given discrete covariates. (*A*) Distribution of latent confounding variable ($Z_{0}$), latent confounding variable selected by positive sign of $V_{1}$ ($Z_{0}^{+1}$), and latent confounding variable selected by negative sign of $V_{1}$ ($Z_{0}^{-1}$). (*B*) The receiver operating characteristic (ROC) curve when using $Z_{0}$ to predict the sign of $V_{1}$. (*C*) The precision–recall (PR) curve when using $Z_{0}$ to predict the sign of $V_{1}$.

From the empirical perspective, we conducted the following experiment to show that the proposed CausalEGM framework can learn to construct such a statistic approximately. In this experiment, 1) $V_{1}$, $V_{2}$,...,$V_{p}$ are i.i.d. binary variables distributed as above. 2) $V_{1}$, $V_{2}$, and $V_{3}$ are used for generating treatment variable and $V_{1}$. 3) $V_{4}$, $V_{5}$ are used for generating outcome variable. Thus $V_{1}$ is the true confounder and $V_{6:p}$ provides independent randomness that is not needed in the generative modeling of treatment and outcome. We set p=15 and the dimension of the latent confounding variable $Z_{0}$ to be 1 (see details in *SI Appendix*, section H). Our numerical results in this setting showed that $Z_{0}$ learned by CausalEGM still follows a standard normal distribution approximately while also preserving most of the information in $V_{1}$ (Fig. 3). We conclude that as long as the high-dimensional covariates assumption holds, the randomness from a sufficient number of nonconfounding covariates can be used for constructing the latent normal variables needed for the conditioning. We also explored the alternative strategy of adding random noise to discrete covariates to directly transform the discrete distribution to a continuous distribution in *SI Appendix*, section H.

### Ablation Study.

Since CausalEGM consists of different modules. It is important to investigate the contribution of each component. First, we test the performance gain brought by the EGM framework. To do this, we removed the G network for reconstructing V and the discriminator network $D_{z}$ for “distribution match” and denoted the model as “CausalEGM∗.” Taking the continuous treatment setting for an example, we note that the performance of CausalEGM without the EGM framework has a noticeable decline in all datasets. The RMSE, MAPE increased by 32.6% to 164.9%, and 14.55% to 377.0%, respectively (Table 3). Such experimental results imply that the adversarial training and the reconstruction error are essential for learning a good low-dimensional representation of the high-dimensional covariates.

**Table 3.** Ablation study on evaluating the contribution of EGM framework

Dataset	Method	RMSE	MAPE
Imbens et al	CausalEGM∗	0.0936±0.0579	0.0434±0.0293
	CausalEGM	0.0706±0.0445	0.0352±0.0210
Sun et al	CausalEGM∗	0.106±0.0473	0.0438±0.0224
	CausalEGM	0.0436±0.0085	0.0180±0.0038
Lee et al	CausalEGM∗	1.28±0.129	0.488±0.0950
	CausalEGM	0.886±0.232	0.426±0.124
Twins	CausalEGM∗	0.0641±0.0252	2.38±6.64
	CausalEGM	0.0242±0.0132	0.499±1.39

CausalEGM∗ represents CausalEGM approach without distribution match in latent space. Each method was run for 10 times and the SD are shown.

Next, we investigate whether the adversarial training for the covariates and the reconstruction for the latent features are necessary. In our model design, adversarial training in latent space is necessary to guarantee the independence of latent variables. The reconstruction of V is also required to ensure the latent features contain all the information possessed by the original covariates. So we designed experiments to quantitatively evaluate the contribution of the adversarial training in covariate space and the reconstruction in latent space. The experiments show that the reconstruction of latent features could benefit the model training and achieve slightly better performance while the adversarial training in covariate space is not that helpful (*SI Appendix*, section I).

### Robustness and Scalability.

We conduct comprehensive experiments to examine the robustness and scalability of CausalEGM (*SI Appendix*, section J). Specifically, we first verify whether CausalEGM is sensitive to the choice of latent feature dimensions, which includes the total dimension of latent space and the dimension of common latent confounders $Z_{0}$. The experimental results show that CausalEGM is quite robust to the choice of latent feature dimensions. For the scalability test, we demonstrate that CausalEGM is capable of handling datasets with a large number of covariates (e.g., >50K) and a large sample size (e.g., >5M) while many competing methods fail.

## Discussion

In this paper, we developed a causal inference model named CausalEMG, which applies a dependency-aware dimension reduction to the high-dimensional covariates and extracts the latent confounding features that are used for covariate adjustment. The proposed EGM framework is shown to be effective in unraveling the dependencies of covariates on treatment and outcome and constructing the generative models for covariates, treatment, and outcome. A wide range of experiments demonstrate the superiority of our approach compared to existing methods. To sum up, CausalEGM is a flexible, scalable, and powerful approach for estimating the causal effect of a variable (e.g., treatment) on another (e.g., outcome), which provides a new perspective to analyze modern observational data in various domains with a large number of covariates and a large sample size.

Several extensions and refinements of the CausalEGM model are left open. First, H(·) function can be further used and adapted to learn the propensity score in both binary and continuous treatment settings. It may benefit the development of new methods where an accurate propensity score model is required. Second, it is worth investigating the interpretable mechanism of latent features. For example, $Z_{2}$ naturally serves as a “latent instrumental variable” where it will only affect the outcome through the treatment variable. How to utilize the latent structure under the EGM framework to help identify (in)valid instruments is an open problem. Third, because of the extreme nonlinearity and complexity of neural network models, mathematically derived statistical properties such as valid CI and convergence rate are almost always missing for deep learning methods. It is helpful to use conformal prediction approaches (43–45) to further study the uncertainty of the estimate.

## Materials and Methods

All the simulated datasets were generated through the generation processes provided by the original papers. Twins dataset was downloaded from https://www.nber.org/research/data/linked-birthinfant-death-cohort-data. The data preprocess was followed by ref. 46. The ACIC 2018 benchmark datasets were downloaded from https://www.synapse.org/#!Synapse:syn11294478/wiki/486304.

## Supplementary Material

Appendix 01 (PDF)

## Data Availability

The project of CausalEGM is maintained at the website https://causalegm.readthedocs.io/ (47), which provides detailed instructions and tutorials. We provide both Pypi Python package CausalEGM (48) and CRAN R package RcausalEGM (49). The source code of CausalEGM is provided at https://github.com/SUwonglab/CausalEGM (50). All study data are included in the article and/or *SI Appendix*.
