# Supplementary material for: An encoding generative modeling approach to dimension reduction and covariate adjustment in causal inference with observational studies
Source: Proc Natl Acad Sci U S A. 2024 May 29;121(23):e2322376121. doi: 10.1073/pnas.2322376121 (PMC11161768; doi:10.1073/pnas.2322376121)
Supplement: Supplementary file 1 — Appendix 01 (PDF) [file pnas.2322376121.sapp.pdf]

Supporting Information for

An encoding generative modeling approach to dimension reduction and covariate adjustment in causal inference with observational studies

Qiao Liu, Zhongren Chen and Wing Hung Wong

Wing Hung Wong.
E-mail: whwong@stanford.edu

This PDF file includes:

Supporting text
Figs. S1 to S4
Tables S1 to S3
SI References

Supporting Information Text

A: Proof of identifiability of $\mu(x)$

$$
\begin{aligned}
\mu(x) &= \mathbb{E}[Y(x)] \\
&= \int \mathbb{E}[f_0(x, Z_y, U_2)|Z_0 = z_0)]p_{Z_0}(z_0)dz_0 \\
&= \int \mathbb{E}[f_0(X, Z_y, U_2)|X = x, Z_0 = z_0]p_{Z_0}(z_0)dz_0 \\
&= \int \mathbb{E}[Y|X = x, Z_0 = z_0)]p_{Z_0}(z_0)dz_0,
\end{aligned} \tag{1}
$$

where the second equality is obtained by the law of total expectation and the third equality is obtained by Assumption 1 ($X \perp\!\!\!\perp Y(x)|Z_0$).

B: Proof of Theorem 1 (Bound of excess risk)

We have two assumptions for the function classes $\mathscr{F}_M$ and $\mathscr{D}_M$.

(**uniformly equi-continuous**) $\mathscr{F}_M$ is uniformly equi-continuous: $\forall \epsilon > 0$, there exists a $\delta > 0$ such that for any $f \in \mathscr{F}_M$ or $f \in \mathscr{D}_M$,

$$|f(x) - f(y)| < \epsilon$$

whenever $d(x, y) < \delta$. Note that we consider the scalar function or each scalar output of a vector function in $\mathscr{F}_M$.

(b-**uniformly bounded**) Assume $\mathscr{F}_M$ and $\mathscr{D}_M$ are b-uniformly bounded: there exists some $b > 0$ such that for any $f \in \mathscr{F}_M$ or $f \in \mathscr{D}_M$, $||f||_\infty \leq b$.

For commonly used neural network models, the first assumption is satisfied as real-valued weights and continuous activation functions are used in neural networks. The second assumption can also satisfied by bounded input domain and regularization on the weights of neural networks.

Next, by using the triangle inequality, we have

$$
\begin{cases}
\mathbb{E}_0[(Y - \hat{f}_{M,n}(X, Z_0, Z_1))^2] \leq \mathbb{E}_n[(Y - \hat{f}_{M,n}(X, Z_0, Z_1))^2] + |(\mathbb{E}_n - \mathbb{E}_0)[(Y - \hat{f}_{M,n}(X, Z_0, Z_1))^2]| \\
\qquad \leq \mathbb{E}_n[(Y - \hat{f}_{M,n}(X, Z_0, Z_1))^2] + \sup_{f \in \mathscr{F}_M} |(\mathbb{E}_n - \mathbb{E}_0)[(Y - f(X, Z_0, Z_1))^2]|, \\
\mathbb{E}_0[(X - \hat{h}_{M,n}(Z_0, Z_2))^2] \leq \mathbb{E}_n[(X - \hat{h}_{M,n}(Z_0, Z_2))^2] + |(\mathbb{E}_n - \mathbb{E}_0)[(X - \hat{h}_{M,n}(Z_0, Z_2))^2]| \\
\qquad \leq \mathbb{E}_n[(X - \hat{h}_{M,n}(Z_0, Z_2))^2] + \sup_{h \in \mathscr{F}_M} |(\mathbb{E}_n - \mathbb{E}_0)[(X - h(Z_0, Z_2))^2]|, \\
\mathbb{E}_0[||V - \hat{g}_{M,n}(\hat{e}_{M,n}(V))||_2^2] \leq \mathbb{E}_n[||V - \hat{g}_{M,n}(\hat{e}_{M,n}(V))||_2^2] + |(\mathbb{E}_n - \mathbb{E}_0)[||V - \hat{g}_{M,n}(\hat{e}_{M,n}(V))||_2^2]| \\
\qquad \leq \mathbb{E}_n[||V - \hat{g}_{M,n}(\hat{e}_{M,n}(V))||_2^2] + \sup_{g,e \in \mathscr{F}_M} |(\mathbb{E}_n - \mathbb{E}_0)[||V - g(e(V))||_2^2]|, \\
d(P_{\hat{e}_{M,n}(V)}, P_{Z^0}; \mathscr{A}_M) \leq d(P_{\hat{e}_{M,n}(V)}, P_{Z_{emp}}; \mathscr{A}_M) + d(P_{Z_{emp}, P_{Z^0}}; \mathscr{A}_M).
\end{cases} \tag{2}
$$

Then by the definition of empirical risk minimizer, we further have

$$
\begin{cases}
\mathbb{E}_n[(Y - \hat{f}_{M,n}(X, Z_0, Z_1))^2] \leq \mathbb{E}_n[(Y - f_M^0(X, Z_0, Z_1))^2], \\
\mathbb{E}_n[(X - \hat{h}_{M,n}(Z_0, Z_2))^2] \leq \mathbb{E}_n[(X - h_M^0(Z_0, Z_2))^2], \\
\mathbb{E}_n[||V - \hat{g}_{M,n}(\hat{e}_{M,n}(V))||_2^2] \leq \mathbb{E}_n[||V - g_{M,n}^0(e_{M,n}^0(V))||_2^2], \\
d(P_{\hat{e}_{M,n}(V)}, P_{Z_{emp}}; \mathscr{A}_M) \leq d(P_{e_M^0(V)}, P_{Z_{emp}}; \mathscr{A}_M)
\end{cases} \tag{3}
$$

where $(f_M^0, h_M^0, e_M^0, g_M^0)$ are the solution of minimizing the true risk $R^0(f, h, e, g)$ and $P_{Z_{emp}}$ is the empirical distribution of Z. Then we use the triangle inequality again, we have

$$
\begin{cases}
\mathbb{E}_n[(Y - f_M^0(X, Z_0, Z_1))^2] \leq \mathbb{E}_0[(Y - f_M^0(X, Z_0, Z_1))^2] + |(\mathbb{E}_n - \mathbb{E}_0)[(Y - f_M^0(X, Z_0, Z_1))^2)]| \\
\qquad \leq \mathbb{E}_0[(Y - f_M^0(X, Z_0, Z_1))^2] + \sup_{f \in \mathscr{F}_M} |(\mathbb{E}_n - \mathbb{E}_0)[(Y - f(X, Z_0, Z_1))^2]|, \\
\mathbb{E}_n[(X - h_M^0(Z_0, Z_2))^2] \leq \mathbb{E}_0[(X - h_M^0(Z_0, Z_2))^2] + |(\mathbb{E}_n - \mathbb{E}_0)[(X - h_M^0(Z_0, Z_2))^2]| \\
\qquad \leq \mathbb{E}_0[(X - h_M^0(Z_0, Z_2))^2] + \sup_{h \in \mathscr{F}_M} |(\mathbb{E}_n - \mathbb{E}_0)[(X - h(Z_0, Z_2))^2]|, \\
\mathbb{E}_n[||V - g_M^0(e_M^0(V))||_2^2] \leq \mathbb{E}_0[||V - g_M^0(e_M^0(V))||_2^2] + |(\mathbb{E}_n - \mathbb{E}_0)[||V - g_M^0(e_M^0(V))||_2^2]| \\
\qquad \leq \mathbb{E}_0[||V - g_M^0(e_M^0(V))||_2^2] + \sup_{g,e \in \mathscr{F}_M} |(\mathbb{E}_n - \mathbb{E}_0)[||V - g(e(V))||_2^2]|, \\
d(P_{e_M^0(V)}, P_{Z_{emp}}; \mathscr{A}_M) \leq d(P_{e_M^0(V)}, P_{Z^0}; \mathscr{A}_M) + d(P_{Z^0}, P_{Z_{emp}}; \mathscr{A}_M).
\end{cases} \tag{4}
$$

Combining all inequations [2,3,4] above, we can then get

$$R^0(\hat{f}_{M,n},\hat{h}_{M,n},\hat{e}_{M,n},\hat{g}_{M,n}) \leq \inf_{f,h,e,g\in\mathscr{F}_M} R^0(f,h,e,g)+\alpha_{M,n}+\beta_{M,n}+\gamma_{M,n}+\zeta_{M,n}. \quad [5]$$

where

$$\begin{cases} \alpha_{M,n} = 2\sup_{f\in\mathscr{F}_M}|(\mathbb{E}_n-\mathbb{E}_0)[(Y-f(X,Z_0,Z_1))^2]|, \\ \beta_{M,n} = 2\sup_{h\in\mathscr{F}_M}|(\mathbb{E}_n-\mathbb{E}_0)[(X-h(Z_0,Z_2))^2]|, \\ \gamma_{M,n} = 2d(P_{Z_{emp}},P_{Z^0};\mathscr{A}_M), \\ \zeta_{M,n} = 2\sup_{g,e\in\mathscr{F}_M}|(\mathbb{E}_n-\mathbb{E}_0)[||V-g(e(V))||_2^2]|. \end{cases} \quad [6]$$

Next, we rewrite the distribution distance measure term from $\gamma_{M,n}$ as

$$d(P_{Z_{emp}},P_{Z^0};\mathscr{A}_M) = \sup_{D\in\mathscr{D}_M}|\frac{1}{n}\sum_{i=1}^{n}\mathbb{1}_{\{D(Z_i)=1\}}-\mathbb{E}_0[\mathbb{1}_{\{D(Z)=1\}}]| = \sup_{D\in\mathscr{D}_M}|(\mathbb{E}_n-\mathbb{E}_0)[\mathbb{1}_{\{D(Z)=1\}}]|, \quad [7]$$

where $\mathscr{D}_M$ is the class of the binary discriminator networks that classifies the class of the measurable sets $\mathscr{A}_M$. Now $\alpha_{M,n}$,$\beta_{M,n}$,$\gamma_{M,n}$, and $\zeta_{M,n}$ all have the same format $2\sup_{F\in\mathscr{F}}|(\mathbb{E}_n-\mathbb{E}_0)F|$ for some function class $\mathscr{F}$. Now for any *b-uniformly bounded* function class $\mathscr{F}$, the uniform law of large numbers states that for all $n\geq 1$ and $\delta\geq 0$, we have:

$$\sup_{F\in\mathscr{F}}|(\mathbb{E}_n-\mathbb{E}_0)F| \leq 2\mathscr{R}_n(\mathscr{F})+\delta, \quad [8]$$

with probability at least $1-2e^{-\frac{n\delta^2}{8b^2}}$. The empirical Rademacher complexity term is defined as

$$\mathscr{R}_n(\mathscr{F}) := \mathbb{E}_\sigma[\sup_{F\in\mathscr{F}}\frac{1}{n}\sum_{i=1}^{n}\sigma_i F(X_i)], \quad [9]$$

where σ_i is *i.i.d.* drawn from the Rademacher distribution with $P(\sigma_i=1)=P(\sigma_i=-1)=\frac{1}{2}$. The Rademacher complexity measures richness of a function class *w.r.t.* a probability distribution.

Note that F is a scalar function (e.g., a loss function of the output for neural network). For example, $F=(Y-f(X,Z_0,Z_1))^2$ in $\alpha_{M,n}$ term. Then we discuss how to further get upper bound of $\alpha_{M,n}$, $\beta_{M,n}$, $\gamma_{M,n}$, and $\zeta_{M,n}$.

Upper bound for $\alpha_{M,n}$, $\beta_{M,n}$. The squared loss functions given the neural network output in $\alpha_{M,n}$ and $\beta_{M,n}$ terms satisfy the Lipschitz condition with bounded inputs, which can be upper bounded by $\mathscr{R}_n(L_\lambda\circ\mathscr{F}_M)$ where L_λ is the λ-Lipschitz squared loss function. Note that the Lipschitz condition is satisfied with bounded domain.

Upper bound for $\gamma_{M,n}$. Let us first denote $\mathscr{H}_M=\{Z\to\mathbb{1}_{\{D(Z)=1\}}:D\in\mathscr{D}_M\}$. Then $\gamma_{M,n}$ can be upper bounded by $\mathscr{R}_n(\mathscr{H}_M)$. If the discriminator (classifier) class $\mathscr{D}_M\subset\{-1,1\}$, then

$$\begin{aligned} \mathscr{R}_n(\mathscr{H}_M) &= \mathbb{E}_\sigma[\sup_{D\in\mathscr{D}_M}\frac{1}{n}\sum_{i=1}^{n}\sigma_i\mathbb{1}_{\{D(Z)=1\}}] \\ &= \mathbb{E}_\sigma[\sup_{D\in\mathscr{D}_M}\frac{1}{n}\sum_{i=1}^{n}\sigma_i\frac{1+D(Z_i)}{2}] \\ &= \mathbb{E}_\sigma[\frac{1}{2n}\sum_{i=1}^{n}\sigma_i+\frac{1}{2}\sup_{D\in\mathscr{D}_M}\frac{1}{n}\sum_{i=1}^{n}\sigma_i D(Z_i)] \\ &= \frac{1}{2}\mathscr{R}_n(\mathscr{D}_M), \end{aligned} \quad [10]$$

where the second to last step follows from the fact that $\mathbb{E}_\sigma[\sigma_i]=0$. If the discriminator (classifier) class $\mathscr{D}_M\subset\{0,1\}$, we could similarly get $\mathscr{R}_n(\mathscr{H}_M)=\mathscr{R}_n(\mathscr{D}_M)$. We choose $\mathscr{D}_M\subset\{0,1\}$ as our default neural network setting.

Upper bound for $\zeta_{M,n}$. We can rewrite $\zeta_{M,n}$ as $\zeta_{M,n}=2\sum_{i=1}^{p}\sup_{g,e\in\mathscr{F}_M}|(\mathbb{E}_n-\mathbb{E}_0)[(V_i-g_i(e(V)))^2]|$ where V_i and $g_i(\cdot)$ represent the i-th dimension of V and $g(\cdot)$, respectively. Then based on the fact $\mathscr{R}_n(\sum_{i=1}^{p}\mathscr{F}_i)\leq\sum_{i=1}^{p}\mathscr{R}_n(\mathscr{F}_i)$ and the function composition $g_i\circ e\in\mathscr{F}_M$, we can get upper bound of the term $\zeta_{M,n}$ by $p\mathscr{R}_n(L_\lambda\circ\mathscr{F}_M)$ since each component in the summation is a squared loss function, which satisfies the Lipschitz condition under bounded domain. In order to bound $\zeta_{M,n}$, we specifically consider the scale of Rademacher complexity of scaler squared loss function and vector squared loss function in bounding $\zeta_{M,n}$.

Finally, the proof is given by the following inequality

$$
\begin{aligned}
&\mathbb{P}(R^0(\hat{f}_{M,n}, \hat{h}_{M,n}, \hat{e}_{M,n}, \hat{g}_{M,n}) - \inf_{f,h,e,g \in \mathscr{F}_M} R^0(f,h,e,g) \leq (8+4p)\mathscr{R}_n(L_\lambda \circ \mathscr{F}_M) + 4\mathscr{R}_n(\mathscr{D}_M) + \delta) \geq \\
&\mathbb{P}(\alpha_{M,n} + \beta_{M,n} + \gamma_{M,n} + \zeta_{M,n} \leq (8+4p)\mathscr{R}_n(L_\lambda \circ \mathscr{F}_M) + 4\mathscr{R}_n(\mathscr{D}_M) + \delta) \geq \\
&\mathbb{P}((4+2p) \sup_{F \in L_\lambda \circ \mathscr{F}_M} |(\mathbb{E}_n - \mathbb{E}_0)F| + 2 \sup_{D \in \mathscr{D}_M} |(\mathbb{E}_n - \mathbb{E}_0)D| \leq (8+4p)\mathscr{R}_n(L_\lambda \circ \mathscr{F}_M) + 4\mathscr{R}_n(\mathscr{D}_M) + \delta)) \geq^{(*)} \\
&\mathbb{P}(\sup_{F \in L_\lambda \circ \mathscr{F}_M} |(\mathbb{E}_n - \mathbb{E}_0)F| \leq 2\mathscr{R}_n(L_\lambda \circ \mathscr{F}_M) + \frac{\delta}{6+2p}, \sup_{D \in \mathscr{D}_M} |(\mathbb{E}_n - \mathbb{E}_0)D| \leq 2\mathscr{R}_n(\mathscr{D}_M) + \frac{\delta}{6+2p}) \geq^{(**)} \\
&\mathbb{P}(\sup_{F \in L_\lambda \circ \mathscr{F}_M} |(\mathbb{E}_n - \mathbb{E}_0)F| \leq 2\mathscr{R}_n(L_\lambda \circ \mathscr{F}_M) + \frac{\delta}{6+2p}) + \mathbb{P}(\sup_{D \in \mathscr{D}_M} |(\mathbb{E}_n - \mathbb{E}_0)D| \leq 2\mathscr{R}_n(\mathscr{D}_M) + \frac{\delta}{6+2p}) - 1 \geq \\
&(1 - 2e^{-\frac{n(\frac{\delta}{6+2p})^2}{8b^2}}) + (1 - 2e^{-\frac{n(\frac{\delta}{6+2p})^2}{8b^2}}) - 1 \geq \\
&1 - 4e^{-\frac{n\delta^2}{32b^2(3+p)^2}}
\end{aligned}
\tag{11}
$$

where $(*)$ is based on a division of δ into two parts with ratio $(4+2p) : 2$ and $(**)$ is based on the fact that $P(A \cup B) = P(A) + P(B) - P(A \cap B) \leq 1$.

Note that if we strictly define $F \in \mathscr{F}_M$ is a scalar output of neural network, such as f, h, e_i and g_i (the i-th output), we can further use Lemma of Talagrand's contraction principal (1) (see its lemma 4.2 and Theorem 10.2) to get upper bound of $\mathscr{R}_n(L_\lambda \circ \mathscr{F}_M)$. If L_λ is $\alpha L^{\alpha-1}$-Lipschitz loss function over bounded domain $[-L, L]$ ($\alpha = 2$ in the squared loss functions), we have $\mathscr{R}_n(L_\lambda \circ \mathscr{F}_M) \leq \alpha L^{\alpha-1} \mathscr{R}_n(\mathscr{F}_M)$.

C: Example for Assumption 2

Assumption 2 is expected to hold with a small δ and the dimension of the covariates V can be effectively reduced. This is illustrated by the following simulation study. Let $V \in \mathcal{V}$ be a continuous random variable that follows a multivariate Gaussian distribution $V \sim N(\boldsymbol{\mu}, \boldsymbol{\Sigma})$ where $\boldsymbol{\mu} \in \mathbb{R}^p$ and $\boldsymbol{\Sigma} \in \mathbb{R}^{p \times p}$. We aim to find encoding function e and generative/decoder function g, which follow the mappings $e : \mathcal{V} \to \mathbb{R}^q$ and $g : \mathbb{R}^q \to \mathcal{V}$ where $q \ll p$. First, we factorize the covariance matrix as

$$
\boldsymbol{\Sigma} = \boldsymbol{U}\boldsymbol{\Lambda}\boldsymbol{U}^T, \tag{12}
$$

where the columns of $\boldsymbol{U}$ form the eigenvectors associated with the eigenvalues in diagonal elements of $\boldsymbol{\Lambda}$. We further sort all the eigenvalues in descending order as $\boldsymbol{\Lambda} = diag(\lambda_1, ..., \lambda_p)$ where $\lambda_i \geq \lambda_j$ for any $i < j$.

By linear transformation, it is easily proven that $\boldsymbol{T} = (\boldsymbol{U}\boldsymbol{\Lambda}^{\frac{1}{2}})^{-1}(\boldsymbol{V} - \boldsymbol{\mu})$ follows a standard multivariate Gaussian distribution where $\boldsymbol{T} \sim N(\boldsymbol{0}, \boldsymbol{I})$. This linear transformation could be considered as the underlying encoding function where a standard Gaussian distribution is present in the latent space. In the dimension reduction scenario, it is expected that a small fraction of eigenvalues in $\boldsymbol{\Sigma}$ could explain the majority of the total variation in V. So we design the following generating process.

We set $p = 50$, $q = 13$, and the diagonal elements of $\boldsymbol{\Lambda}$ to be

$$
\lambda_i = \begin{cases} 5 - \frac{1}{9}(i-1), & i \leq 10, \\ 0.1 - \frac{1}{400}(i-11), & 11 \leq i \leq 50 \end{cases} \tag{13}
$$

where the first 13 principle components can explain 95.96% of the variation contained in V. To generate V, the mean vector $\boldsymbol{\mu}$ is sampled from a uniform distribution $\mu_i \sim U(-1, 1)$, the covariance matrix $\boldsymbol{\Sigma}$ is constructed by Equation (12) where the columns of $\boldsymbol{U}$ are a set of random orthonormal basis. To construct the features from V for predicting treatment X and outcome Y, we set the three components $e_0^0(V)$, $e_1^0(V)$, and $e_2^0(V)$ in the encoder network e as follows

$$
\begin{cases} e_0^0(V) = (t_8(V) + t_{11}(V))/\sqrt{2}, \\ e_1^0(V) = (t_9(V) + \sum_{i=12}^{20} t_i(V))/\sqrt{10}, \\ e_2^0(V) = (t_{10}(V) + \sum_{i=22}^{30} t_i(V))/\sqrt{10}. \end{cases} \tag{14}
$$

where $t_i(V)$ denotes that i^{th} element of the linear transformation $\boldsymbol{T} = (\boldsymbol{U}\boldsymbol{\Lambda}^{\frac{1}{2}})^{-1}(\boldsymbol{V} - \boldsymbol{\mu})$. It is easily proven that $e_k^0(V) \sim N(0, 1)$ for $k \in \{1, 2, 3\}$, which satisfies the independent normal distribution in latent space. The treatment and outcome can then be generated based on the features of V as

$$
\begin{cases} Y = f(X, e_0^0(V), e_1^0(V)) + \epsilon_1, \\ X = h(e_0^0(V), e_2^0(V)) + \epsilon_2, \end{cases} \tag{15}
$$

where $e_0^0(V)$, $e_1^0(V)$, and $e_2^0(V)$ can be considered as the constructed features from V for predicting X and Y. For implementation, we set the first three parts of encoder e network to be the fixed functions, $e_0^0(\cdot)$, $e_1^0(\cdot)$, and $e_2^0(\cdot)$. The fourth part of encoder e is trainable, which is set to be 10-dimensional. According to the Principal Component Analysis (PCA) (2), the theoretical optimal reconstruction error using a q-dimensional feature is

$$\mathcal{L}_{rec} = \sum_{i=q+1}^{p} \lambda_i. \quad [16]$$

In the above simulation example, $\mathcal{L}_{rec} = 1.907$. Then we generate $N = 50000$ $i.i.d$ samples of V, and then use the data to train the above partially fixed encoder-decoder model. To avoid overfitting of neural nets, we additionally generate 10000 hold-out samples of V. As shown in Fig S1, the empirical reconstruction error of the held-out data reaches the minimum (2.339) at iteration 109600. In this simulation, δ in assumption 2 can be as small as 0.432, which only occupies less than 1% of all variation contained in V ($\sum_{i=1}^{p} \lambda_i$).

D: Proof of Theorem 2 (Consistency)

By Borel–Cantelli lemma and Theorem 1, for any $\delta' > 0$, we would have

$$(\dagger): R^0(f^*, h^*, e^*, g^*) \leq R^0(f, h, e, g) + \delta', \quad [17]$$

where (f^*, h^*, e^*, g^*) are the limit point of the MER. In particular, we can choose δ' to be the same δ as in Assumption 2.

During the CausalEGM model training, we aim to optimize (F, H, E, G) networks to approximate the underlying true functions f^0, h^0, e^0, and g^0. Then suppose

$$\begin{cases} X = h^0(Z_0, Z_2) + \epsilon_2, \\ Y = f^0(X, Z_0, Z_1) + \epsilon_1, \\ V = g^0(Z), \end{cases} \quad [18]$$

where $(Z_0, Z_1, Z_2) = (e_0^0(V), e_1^0(V), e_2^0(V))$, $\mathbb{E}[\epsilon_1] = \mathbb{E}[\epsilon_2] = 0$, $\text{Var}(\epsilon_1) = \sigma_1^2$, $\text{Var}(\epsilon_2) = \sigma_2^2$, $\epsilon_2 \perp\!\!\!\perp (Z_0, Z_2)$, $\epsilon_1 \perp\!\!\!\perp (Z_0, Z_1, X)$. $(e_0^0, e_1^0, e_2^0, e_3^0)$ denotes the four components of the encoder function e^0. We want the latent variable $Z = (Z_0, Z_1, Z_2, Z_3)$ to have a desired distribution (e.g., standard normal distribution).

Based on equations 18, for any (f, h, e, g), we have

$$\begin{aligned} R^0(f, h, e, g) &= \mathbb{E}_0[(Y - f(X, Z_0, Z_1))^2] + \mathbb{E}_0[(X - h(Z_0, Z_2))^2] \\ &\quad + d(P_{Z^0}, P_{e(V)}; \mathscr{A}_m) + \mathbb{E}_0[||V - g(e(V))||_2^2] \\ &= \mathbb{E}_0[((f^0 - f)(X, Z_0, Z_1))^2] + \sigma_1^2 + \mathbb{E}_0[((h^0 - h)(Z_0, Z_2))^2] + \sigma_2^2 \\ &\quad + d(P_{Z^0}, P_{e(V)}; \mathscr{A}_M) + \mathbb{E}_0[||(V - g(e(V)))||_2^2]. \end{aligned} \quad [19]$$

Let $e_3', g' = \underset{e_3, g}{\text{argmin}}\{d(P_{Z^0}, P_{(e_0^0, e_1^0, e_2^0, e_3)(V))}; \mathscr{A}_m) + \mathbb{E}_0[||V - g(e_0^0, e_1^0, e_2^0, e_3)(V))||_2^2]\}$, $f = f^0, h = h^0, e = (e_0^0, e_1^0, e_2^0, e_3')$ and $g = g'$. We substitute into the right-hand side of $(\dagger)$ and get

$$\begin{aligned} R^0(f^*, h^*, e^*, g^*) &\leq \sigma_1^2 + \sigma_2^2 + d(P_{Z^0}, P_{(e_0^0, e_1^0, e_2^0, e_3')(V)}; \mathscr{A}_M) + \mathbb{E}_0[||V - g'((e_0^0, e_1^0, e_2^0, e_3')(V))||_2^2] + \delta \\ &\leq \sigma_1^2 + \sigma_2^2 + d(P_{Z^0}, P_{(e_0^0, e_1^0, e_2^0, \tilde{e}_3)(V)}; \mathscr{A}_M) + \mathbb{E}_0[||V - \tilde{g}((e_0^0, e_1^0, e_2^0, \tilde{e}_3)(V))||_2^2] + \delta \\ &\overset{Asm.2}{\leq} \sigma_1^2 + \sigma_2^2 + \mathbb{E}_0[||V - g^*(e^*(V))||_2^2] + 2\delta. \end{aligned} \quad [20]$$

Note that the last inequality in 20 holds based on Assumption 2 in the main text where we have 1) $(e_0^0, e_1^0, e_2^0, \tilde{e}_3) \overset{\mathcal{D}}{=} Z^0$ and 2) $\mathbb{E}_0[||V - \tilde{g}((e_0^0, e_1^0, e_2^0, \tilde{e}_3)(V))||_2^2] \leq \mathbb{E}_0[||V - g((e)(V))||_2^2] + \delta$ for any e and g. Here we set $e = e^*$ and $g = g^*$.

On the other hand, according to equation 19 we have

$$\begin{aligned} R^0(f^*, h^*, e^*, g^*) =& \mathbb{E}_0[((f^0 - f^*)(X, Z_0, Z_1))^2] + \sigma_1^2 + \mathbb{E}_0[((h^0 - h^*)(Z_0, Z_2))^2] + \sigma_2^2 + \\ & d(P_{Z^0}, P_{e^*(V)}; \mathscr{A}_M) + \mathbb{E}_0[||V - g^*(e^*(V))||_2^2]. \end{aligned} \quad [21]$$

Combine inequation 20 and equation 21, we finally have

$$\mathbb{E}_0[((f^0 - f^*)(X, Z_0, Z_1))^2] + \mathbb{E}_0[((h^0 - h^*)(Z_0, Z_2))^2] + d(P_{Z^0}, P_{e^*(V)}; \mathscr{A}_M) \leq 2\delta. \quad [22]$$

E: Details of Datasets

Imbens et al. We follow a similar data-generating process as in (3) and (4) as follows: let V_1, V_2,...,V_p be i.i.d. unit exponential random variables, $Z_0 = V_1$, $Z_1 = V_2$, $Z_2 = V_3$, $X|V \sim exp(Z_0 + Z_1)$, and $Y(x)|V \sim N(x + (Z_0 + Z_2)exp(-x(Z_0 + Z_2), 1)$. Then the dose-response function can be obtained by integration w.r.t the covariates V: $\mu(x) = x + \frac{2}{(1+x)^3}$.

Sun et al. We generate a synthetic dataset using a similar data generating process described in (5). with some modifications to fit for continuous treatment. Specifically, we let $V_1, ..., V_p \overset{iid}{\sim} N(0,1)$ and define $f_1(u) = -2sin(2u)$, $f_2(u) = u^2 - \frac{1}{3}$, $f_3(u) = u - \frac{1}{2}$, $f_4(u) = cos(u)$, $f_5(u) = u^2$ and $f_6(u) = u$. We then generate the treatment to be $X \sim N(\sum_{i=1}^4 f_i(V_i), 1)$ and the outcome to be $Y \sim N(X + f_3(V_1) + f_4(V_2) + f_5(V_5) + f_6(V_6), 1)$. Then the dose-response function can be obtained by integration w.r.t the covariates V: $\mu(x) = x + 0.5 + e^{-0.5}$..

Lee et al. We followed a similar data generation process in (6) as follows: let $\epsilon_1 \sim N(0,1)$, $\epsilon_2 \sim N(0,1)$. The covariates are generated by $V = (V_1, ..., V_p)^{'} \sim N(0, \Sigma)$ where $diag(\Sigma) = 1$ and $\Sigma_{i,j} = 0.5$ for $|i-j| = 1$. The treatment is generated by $X = \Phi(3V^{'}\theta) + 0.75\epsilon_1 - 0.5$ where $\theta_j = 1/j^2$. The outcome is generated by $Y = 1.2X + 1.2V^{'}\theta + X^3 + XV_1 + \epsilon_2$. The dose-response function is $\mu(x) = 1.2x + x^3$.

Twins. This dataset contains data of 71,345 twins, including their weights (used as treatment), mortality, and 50 other covariates (so $p = 50$) derived from all births in the USA between 1989-1991. Similar to (7), we first filtered the data by limiting the weight to be less than 2 kilograms. 4821 pairs of twins were kept for further analysis. We set the weights as the continuous treatment variable. We then simulate the risk of death (outcome) under a model in which higher weight leads to a lower death rate in general. Let Y be the Bernoulli variable where $Y = 1$ indicates death and R be the death risk that depends on the covariates. We simulate the outcome as $Y(x) \sim Bernoulli(R(x))$, $R(x) = -\frac{2}{1+e^{-3X}} + v\gamma + \epsilon$ where $\gamma \in \mathbb{R}^{p\times 1}$ and $\gamma_i \sim N(0, 0.025^2)$, $\epsilon \sim N(0, 0.25^2)$. Taking the response variable as R instead of Y, the ADRF is then: $\mu(x) = -\frac{2}{1+e^{-3x}} + \mathbb{E}[V_i \cdot \gamma]$.

ACIC 2018. For binary treatment settings, we downloaded the datasets from the 2018 Atlantic Causal Inference Conference (ACIC) competition. This dataset utilizes the Linked Births and Infant Deaths Database (LBIDD) based on real-world medical measurements collected from (8). The LBIDD data is semi-synthetic where 117 measured covariates are given, and the treatment and outcome are simulated based on different data-generating processes. We chose nine datasets by selecting the most complicated generation process (e.g., the highest degree of generation function) with sample sizes ranging from 1,000 to 50,000. The details for the datasets used in the study are provided in Table S1.

F: Baseline Methods

For the continuous treatment setting, three different baselines were used.

Ordinary Least Squares regression (OLS). OLS first fit a linear regression model for $Y|(X, V)$. For each value of treatment x, the estimated ADRF is then $\frac{1}{n}\sum_i^n ls(x, v_i)$ where ls is the fitted linear model.

Regression Prediction Estimator (REG). See (9–11). The *prima facie* estimator is an estimator that regresses the outcome on the treatment without considering covariates. REG generalizes the notion of *prima facie* estimator. It takes the covariates into account when doing regression. Unlike OLS, REG fits a quadratic ADRF: $Y(x) = \alpha_0 + \alpha_1 x + \alpha_2 x^2$.

Double Debiased Machine Learning Estimator (DML). See (6). DML is a kernel-based machine learning approach that combines a doubly moment function and cross-fitting. Various machine learning methods can be used to estimate the conditional expectation function and conditional density. We used "Lasso", "random forest", and "neural network" provided by the DML toolkit as three variants, denoted as DML(lasso), DML(rf) and DML(nn).

For the binary treatment setting, six baselines were introduced.

CFR. See (12). This method estimated the individual treatment effect (ITE) by utilizing neural networks to learn the low-dimensional representation for covariates and two outcome functions, respectively. An integral probability metric was further introduced to control the balance of distributions in the treated and control group. We use the two variants of CFR for comparison, which are referred to as TAENET and CFRNET.

Dragonnet. See (13). This method used a three-head architecture, which contains a two-head architecture for outcome estimation and a one-head architecture for propensity score estimation. It is noted that Dragonnet uses essentially the same architecture as CFR if the propensity-score head is removed.

CEVAE. See (14). This method is a variational autoencoder-based method for estimating the treatment effect where the above CFR architecture (12) was used in the inference network and the latent variables were set to be multivariate normal distribution.

GANITE. See (15). This method exploited a generative adversarial network (GAN) model for generating the counterfactual outcome through adversarial training.

CausalForest. .See (16). This method built random forests to estimate the heterogeneous treatment effect that is applicable in binary treatment settings. Causalforest is an ensemble method that consists of multiple causal trees.

SDRcausal. See (17). This method uses linear sufficient dimension reduction for both treatment and outcome model. The propensity score is modeled as $1/(1+exp(-\eta(\alpha^T V)))$ and the potential outcome is modeled as $Y_k = m_k(\beta_T x) + \epsilon_k (k=0,1)$. α, β_0, and β_1 are the projection matrix/vector in the sufficient dimension reduction to be learned. Note that SDRcausal implements several different variants from the original paper and we always choose the best result to report.

G: Comparison with Sufficient Dimension Reduction

We made a comprehensive comparison of CausalEGM with SDRcausal under experimental settings either satisfying or violating the SDR assumption. CausalEGM shows great improvement over SDRcausal, especially in the nonlinear dataset.

We used the default hyperparameters of CausalEGM and used the default parameters provided by SDRcausal software. Note that SDRcausal implements several different variants from the original paper and we always chose the best result to report. We used two datasets (linear SDR and nonlinear SDR) for benchmark comparison.

1) The simulation dataset was obtained from the original SDRcausal paper, which satisfies the SDR assumption (linear). 6 covariates were considered. $V_1 \sim N(1,1), V_2 \sim N(0,1), V_3 \sim Bernoulli(0.5+0.005V_2), V_4 \sim 0.015V_1 + U(-0.5,0.5), V_5 \sim Bernoulli(0.4+0.2V_4), V_6 = 0.004V_2 + 0.15V_3 + 0.05V_4 + N(0,1)$. $\alpha = (-0.27, 0.2, -0.15, 0.05, 0.15, -0.1)^T$, $\beta_0 = (1,1,0,0,0,0)^T$, $\beta_1 = (1,-1,1,-2,-1.5,0.5)^T$ The generation process is

$$\begin{cases} Y_0 = \beta_0^T V + N(0,0.2), \\ Y_1 = 0.7(\beta_1^T V)^2 + \sin(\beta_1^T V) + N(0,0.5), \\ P(X=1|V) = 1/(1+exp(-\alpha^T V)). \end{cases} \quad [23]$$

2) We replaced the linear SDR term with different nonlinear functions, such as polynomial and trigonometric terms, in the generation process of the first dataset, which is represented as

$$\begin{cases} Y_0 = m_0(V) + N(0,0.2), \\ Y_1 = 0.7(m_1(V))^2 + \sin(m_1(V)) + N(0,0.5), \\ P(X=1|V) = 1/(1+exp(-m_x(V))), \end{cases} \quad [24]$$

where $m_0(V) = \sin(V_1) + \cos(V_2) + \exp(V_3) + V_4^3 + \sin(V_5 V_6)$, $m_1(V) = \cos(V_1 V_2) + V_3^2 + \exp(V_4) + \sin(V_5) + \cos(V_6)$, $m_x(V) = V_1 V_2 + V_3 X_4^3 + V_5^2 V_6$. The linear SDR does not work in this case while the deep learning model CausalEGM can capture the nonlinearity in this dataset.

Then, we run CausalEGM and SDRcausal for independent 10 times for each dataset with different sample size. CausalEGM achieves significantly better performance than SDRcausal with default settings in both datasets with different sample size (Table S2). For the first dataset, if we initiate the linear SDR parameters $(\alpha, \beta_0, \beta_1)$ to be the ground truth parameters in the generation process, then SDRcausal could achieve slightly better performance than CausalEGM, which is an expected result as this data set satisfied the linearity assumption and SDRcausal should be more efficient if the optimization is solved correctly. Beside, we also found SDRcausal is not scalable as it fails to run on the ACIC datasets used in our paper when the sample size is larger than 10k.

H: Impact of discrete covariates

We designed a simulation dataset with the most extremely discrete covariates as follows. Let V_1, V_2,...,V_n be a *i.i.d.* distribution where $P(V_i = 1) = P(V_i = -1) = 1/2$. $\alpha = (1,1,-1)^T$, $\beta = (1,-1,1)^T$. The generation process is

$$\begin{cases} Y_0 = \beta^T V + N(0,0.01^2), \\ Y_1 = 0.3(\beta^T V)^2 + N(0,0.01^2), \\ P(X=1|V) = 1/(1+exp(-\alpha^T V)). \end{cases} \quad [25]$$

We set $n = 15$ and the sample size to be $10,000$, and the latent dimensions to be $(1,2,2,5)$, we run CausalEGM on the above simulation dataset.

From the theoretical perspective, under the condition that there are enough covariates that are independent of both treatment and response, we can construct an approximated standard normal latent distribution even when the confounders are discrete variables. Let us consider a general case where $P(V_i = 1) = p$ and $P(V_i = -1) = 1-p$ $(0 < p < 1)$, $S = \sum_i^n V_i$, then $P(S=k) = \binom{n}{\frac{n+k}{2}} p^{\frac{n+k}{2}} (1-p)^{\frac{n-k}{2}}$ where $k \in \{-n, -n+2, ..., n-2, n\}$. $E(V_i) = 2p-1$ and $Var(V_i) = 4p(1-p)$. According to central limit theorem, we have $\frac{S-n(2p-1)}{\sqrt{4np(1-p)}} \sim N(0,1)$ when n is sufficiently large. In the above example where $p = 1/2$, we can construct a statistic $W = V_1 \frac{|\sum_{i=2}^n V_i|}{\sqrt{n-1}}$ to satisfy the following two conditions 1) approximate $N(0,1)$. 2) W contains all the information in the confounder as V_1 can be covered by taking the sign of W.

Furthermore, we explored a natural strategy by adding random noise with a moderate level to the discrete covariates to make them more continuous but still distinguishable. We adapted the first dataset from the SI Appendix where V_i has the *i.i.d.* distribution $V_i \sim Bernoulli(0.5)$ and $i = 1, ..., 10$. The generation process is

$$\begin{cases} Y_0 = \beta_0^T V_y + N(0, 0.2), \\ Y_1 = 0.7(\beta_1^T V_y)^2 + \sin(\beta_1^T V_y) + N(0, 0.5), \\ P(X = 1|V) = 1/(1 + exp(-\alpha^T V_{1:6})). \end{cases} \quad [26]$$

where V_y is the concatenation of $V_{1:K}$ and $V_{(K+5):10}$. Then we varied K to set different number of confounders. It is observed that adding a Gaussian noise ($N(0, 0.2^2)$) could slightly improve the model performance with different K (Figure S4). To conclude, we can add Gaussian noise to the discrete covariates to mitigate the discreteness if the number of covariates is not big. However, adding noise to all covariates maybe harmful to the model as the total variance could accumulate if the latent confounding variable is generated through the combination of many covariates. In the case of high-dimensional covariates, users shall directly run CausalEGM without adding any noise.

I: Ablation Studies

We conducted detailed experiments to investigate whether the adversarial training in covariate space and the reconstruction error in latent space are necessary. In our model design, adversarial training in latent space is necessary to guarantee the independence of latent variables. The reconstruction of V is also required for ensuring the latent features contain all the information possessed by the original covariates. So we designed experiments using two datasets to quantitatively evaluate the contribution of the adversarial training in covariate space and the reconstruction in latent space. As shown in Table S3, the reconstruction of latent features could help benefit the model training and achieve slightly better performance. Using the adversarial training in covariate space might not improve the model training as the distribution matching in high-dimensional space might be difficult.

J: Robustness and Scalability

To demonstrate the robustness and scalability of CausalEGM, we designed a series of experiments as follows. For the robustness analysis, it is important to evaluate how the dimension for latent features Z will affect the performance of CausalEGM model. We focus on both the total dimension of latent feature Z and also the dimension of the common latent features Z_0 that affect both treatment and outcome. For continuous experiments, we choose Hiranos and Imbens dataset for example. On the one hand, the dimension for Z_0, Z_1, Z_2, Z_3 is set to be $\{(k, k, k, 7k)\|k = 1, 2, ..., 5\}$ where $k = 1$ is used as default in the main result. On the other hand, we set the dimension for Z_0 to be ranging from 1 to 10 while dimensions of other latent features ($Z_i(i = 1, 2, 3)$) are fixed to $(1, 1, 7)$, respectively. It is noted that the performance has a small fluctuation by varying the dimension of either total latent features or only common latent features (Figure S2A-B). We use similar settings in the binary experiment for robustness analysis. We chose a dataset from LBIDD with a sample size equal to 1000 for instance. On the one hand, the dimension for Z_0, Z_1, Z_2, Z_3 is set to be $\{(k, k, 2k, 2k)|k = 1, 2, ..., 5\}$ where $k = 3$ is used as default. On the other hand, we set the dimension for Z_0 to be ranging from 1 to 10 while dimensions of other latent features ($Z_i(i = 1, 2, 3)$) are fixed to $(3, 6, 6)$. It is observed that the performance does not change significantly by varying the dimension of latent features (Figure S2C-D). Such experiments in both continuous and binary treatment settings demonstrate the robustness of CausalEGM in terms of choosing the latent dimensions.

For the scalability analysis, we are interested in 1) whether CausalEGM can handle datasets with large sample sizes; and 2) whether CausalEGM can handle datasets with a large number of covariates. We designed the following experiments to test the scalability of CausalEGM. For the continuous treatment experiment, we selected Hirano and Imbens dataset. We first change the number of covariates from 50 to 10000 while the sample size is 10000. Note that only OLS, DML(lasso) and CausalEGM can handle covariates more than 1000 while other comparison methods failed (Figure S3 A). Next, we fix the number of covariates to 100 while changing the sample size from 10^3 to 10^6. Only OLS, REG, DML(lasso), and CausalEGM are able to handle large sample size 10^6 (Figure S3 B). Except for a small sample size situation (e.g., 1000) where CausalEGM achieves comparable performance compared to DML(nn) and DML(rf), CausalEGM consistently outperforms all comparison methods by either changing the number of covariates or sample size. Similarly, for the binary treatment experiment, we chose one of the largest dataset from LBIDD with a sample size equal to 50000. For such a semi-synthetic dataset, we increase the number of covariates by adding new covariates that are linear combinations of existing covariates where the combination coefficients follow the standard normal distribution. We increase the sample size by augmenting the data by randomly repeating the existing samples. We tested the performance of CausalEGM and the best baseline method CausalForest. We first change the number of covariates from 500 to 50000 while the sample size is 50000. Note that the performance of CausalForest first increases a little and then decreases while CausalEGM is generally more robust when changing the number of covariates (Figure S3 C). Next, we fix the number of covariates to the original 177 while changing the sample size from 50000 to 5000000. Note that CausalForest failed when the sample size increases to 1 million while CausalForest is capable of handling extremely large datasets with more than 5 million samples (Figure S3D). Note that we benchmarked all methods using the Stanford Sherlock computing cluster where the memory usage for each method is limited to 50 GB and running time is limited to 7 days in the scalability experiments. To sum up, CausalEGM can handle significantly larger datasets than CausalForest.

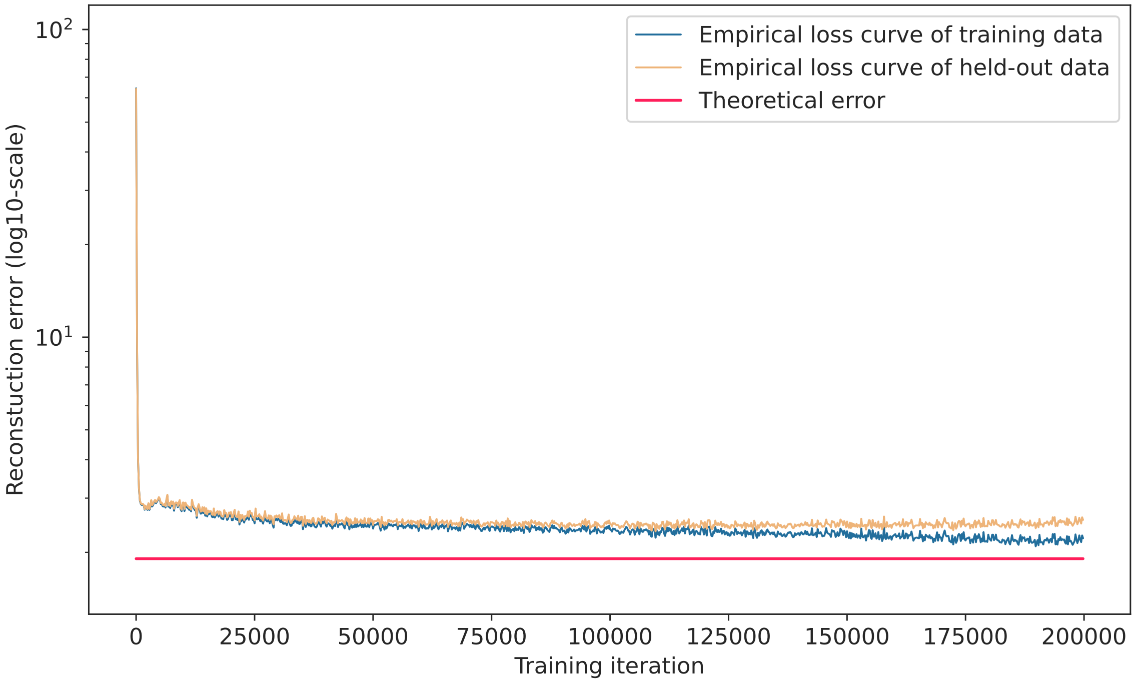

Fig. S1. The simulation experiment for verifying the assumption 2

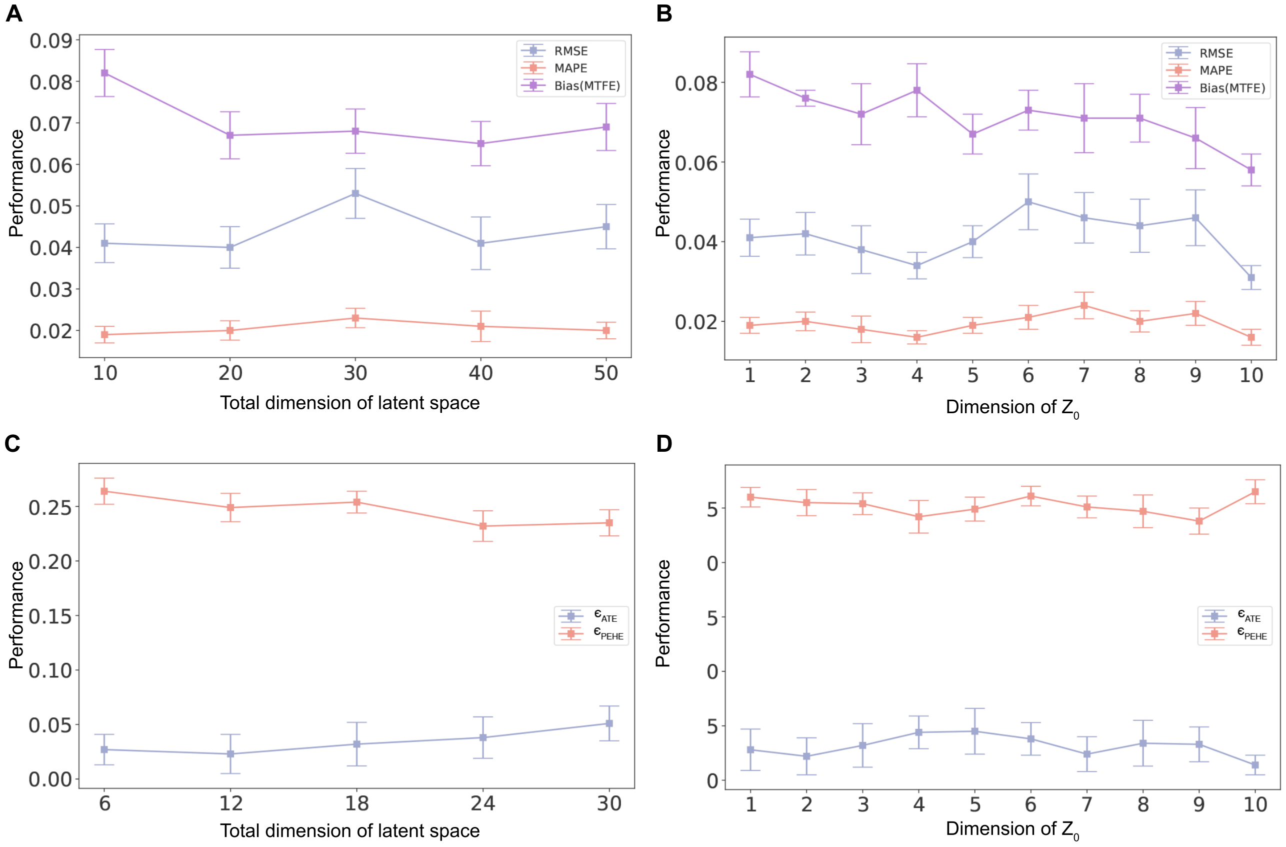

Fig. S2. Robustness of latent dimensions in CausalEGM model. The error bar denotes the standard deviation of 10 independent runs. (A) Performance when the total dimension of latent features varies from 10 to 50 in Hiranos and Imbens dataset. (B) Performance when the dimension of Z_0 varies from 1 to 10 while dimensions of other $Z_i(i = 1, 2, 3)$ are fixed in Hiranos and Imbens dataset. (C) Performance when the total dimension of latent features varies from 6 to 30 in a Twins dataset. (D) Performance when the dimension of Z_0 varies from 1 to 10 while dimensions of other $Z_i(i = 1, 2, 3)$ are fixed in the Twins dataset.

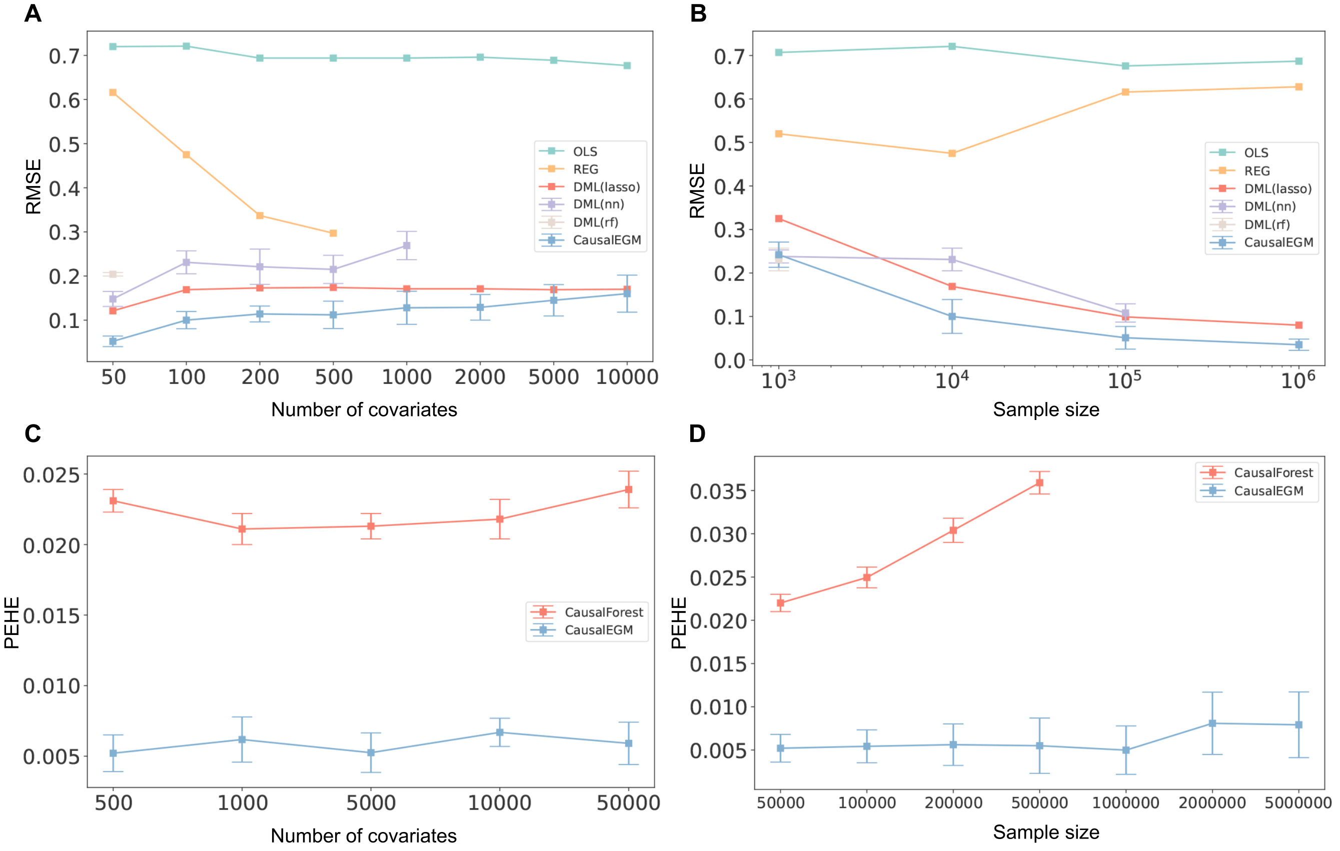

Fig. S3. Scalability of CausalEGM model in terms of covariates and sample size. The error bar denotes the standard deviation of 10 independent runs. (A) Performance when changing the number of covariates from 50 to 10000 in Hirano and Imbens dataset. (B) Performance when changing the sample size from 10^3 to 10^6 in Hirano and Imbens dataset. (C) Performance when changing the number of covariates from 500 to 50000 in the Twins dataset. (D) Performance when changing the sample size from 50000 to 5000000 in the Twins dataset.

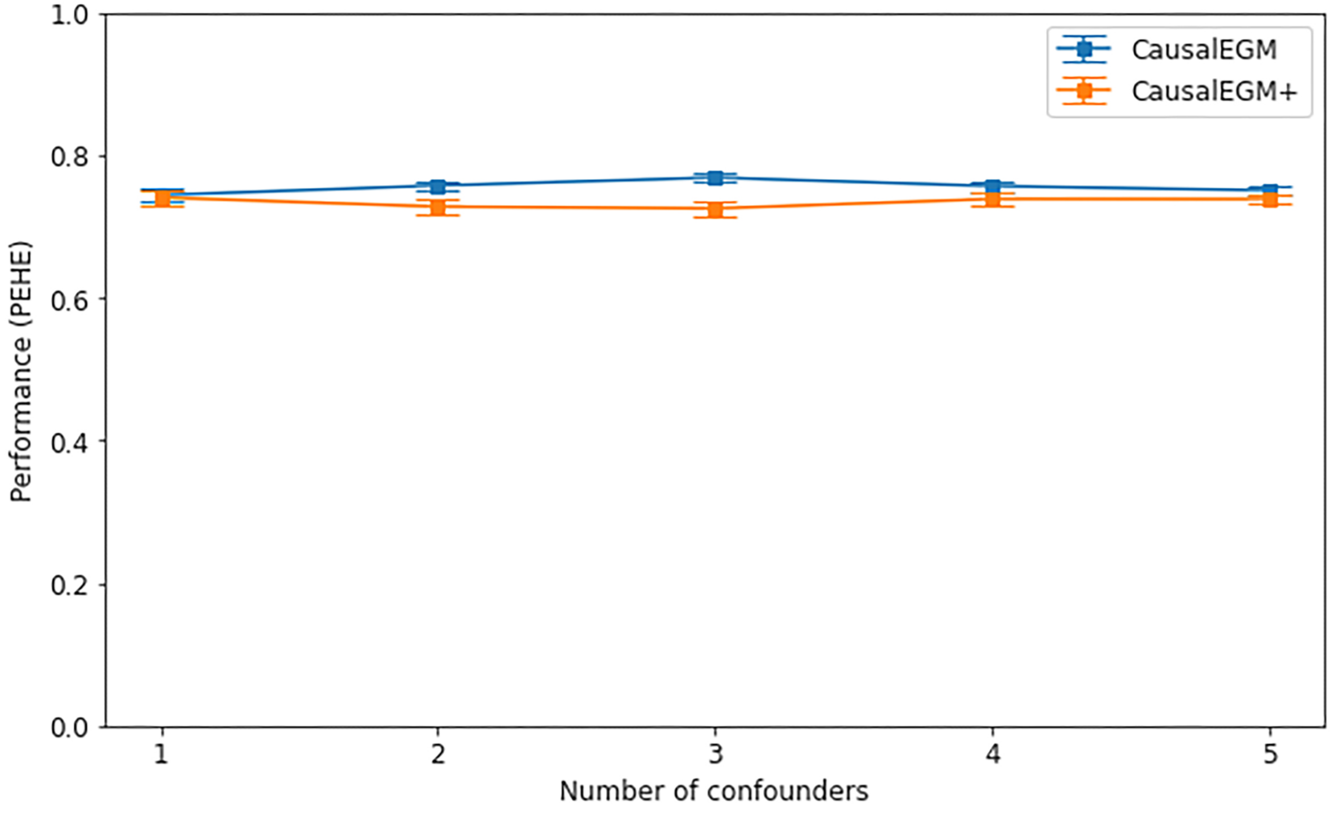

Fig. S4. Model performance with different number of confounders using the original discrete covariates (CausalEGM) or adding Gaussian noise to the discrete covariates (CausalEGM+).

Table S1. Details of binary treatment datasets from ACIC 2018 used in this study. Each dataset has a unique ufid series number.

Sample size	Ufid	Treated percentage	True ATE
Datasets-1k	629e3d2c63914e45b227cc913c09cebe	36.76%	0.006208
	35524a031525484dab3b06f3728c708e	10.01%	0.513605
	a957b431a74a43a0bb7cc52e1c84c8ad	46.44%	6.373162
Datasets-10k	71f29913f174456e9fe2727b1b86b8b3	57.98%	9.243389
	fda655aeb8644c9db5c543ed9d1006ad	22.05%	-0.0566
	05fdeea9fcb64b3885e6ebfb85b4ce90	12.08%	0.025841
Datasets-50k	1c565ac309074f178a377c2759333209	14.48%	-0.468357
	b73beac2f4c349fb981880399d4c88a6	18.79%	-0.049168
	d5bd8e4814904c58a79d7cdcd7c2a1bb	54.50%	-0.296505

Table S2. The comparison of CausalEGM and SDRcausal under both linear and nolinear SDR settings with three different sample sizes (2k, 5k, 10k). SDRcausal(DF) denotes using the default initial values in the model fitting. SDRcausal(GT) denoted directly giving the ground true parameter values as the initial values in the fitting of the model. Each method was run 10 times and the standard deviations are shown. The best performance is marked in bold.

Metric	Setting	SDRcausal(DF)	SDRcausal(GT)	CausalEGM
ϵ_{ATE}	Linear SDR	0.537 ± 0.070	$\mathbf{0.034}$	0.045 ± 0.027
		0.415 ± 0.044	0.083	$\mathbf{0.053 \pm 0.065}$
		0.331 ± 0.044	$\mathbf{0.038}$	0.070 ± 0.043
	Nolinear SDR	0.204 ± 0.131	-	$\mathbf{0.080 \pm 0.043}$
		0.176 ± 0.122	-	$\mathbf{0.096 \pm 0.084}$
		0.200 ± 0.185	-	$\mathbf{0.080 \pm 0.065}$
ϵ_{PEHE}	Linear SDR	14.49 ± 1.34	$\mathbf{0.684}$	0.918 ± 0.037
		13.55 ± 1.39	$\mathbf{0.708}$	0.822 ± 0.014
		13.31 ± 1.08	$\mathbf{0.711}$	0.811 ± 0.013
	Nonlinear SDR	11.52 ± 0.58	-	$\mathbf{1.39 \pm 0.07}$
		11.13 ± 0.67	-	$\mathbf{1.467 \pm 0.086}$
		11.95 ± 1.08	-	$\mathbf{1.307 \pm 0.071}$

Table S3. Experiments on Robustness of loss for continuous treatments. The indicators in the second column denotes whether we use the adversarial training in covariate space (V-GAN) and the reconstruction term for latent features (Z-Rec). Each method was run for five times independently and the standard deviations are shown.

Dataset	(V-GAN, Z-Rec)	RMSE	MAPE	Bias(MTFE)
Hiranos and Imbens	(1,1)	0.0906 ± 0.0270	0.0439 ± 0.0116	0.104 ± 0.200
	(0,1)	$\mathbf{0.0727 \pm 0.0451}$	$\mathbf{0.0345 \pm 0.0190}$	$\mathbf{0.0890 \pm 0.0230}$
	(1,0)	0.0845 ± 0.0321	0.0401 ± 0.00984	0.0940 ± 0.0355
	(0,0)	0.0784 ± 0.0363	0.0371 ± 0.0163	0.103 ± 0.0352
Sun et al	(1,1)	0.0567 ± 0.0299	0.0219 ± 0.0134	0.0280 ± 0.0282
	(0,1)	$\mathbf{0.0436 \pm 0.00857}$	$\mathbf{0.0180 \pm 0.00388}$	$\mathbf{0.0230 \pm 0.0116}$
	(1,0)	0.0592 ± 0.0202	0.0227 ± 0.00826	0.03002 ± 0.0267
	(0,0)	0.0622 ± 0.0322	0.0234 ± 0.0124	0.0266 ± 0.0252

References

1. M Mohri, A Rostamizadeh, A Talwalkar, *Foundations of machine learning.* (MIT press), (2018).
2. K Pearson, Liii. on lines and planes of closest fit to systems of points in space. *The London, Edinburgh, Dublin philosophical magazine journal science* **2**, 559–572 (1901).
3. K Hirano, GW Imbens, The propensity score with continuous treatments. *Appl. Bayesian modeling causal inference from incomplete-data perspectives* **226164**, 73–84 (2004).
4. EE Moodie, DA Stephens, Estimation of dose–response functions for longitudinal data using the generalised propensity score. *Stat. methods medical research* **21**, 149–166 (2012).
5. W Sun, P Wang, D Yin, J Yang, Y Chang, Causal inference via sparse additive models with application to online advertising in *Twenty-Ninth AAAI Conference on Artificial Intelligence.* (2015).
6. K Colangelo, YY Lee, Double debiased machine learning nonparametric inference with continuous treatments. *arXiv preprint arXiv:2004.03036* (2020).
7. Y Li, et al., Continuous treatment effect estimation via generative adversarial de-confounding in *Proceedings of the 2020 KDD Workshop on Causal Discovery*, Proceedings of Machine Learning Research. (PMLR), Vol. 127, pp. 4–22 (2020).
8. E Karavani, Y Shimoni, C Yanover, Ibm causal inference benchmarking framework (2018).
9. J Schafer, D Galagate, Causal inference with a continuous treatment and outcome: alternative estimators for parametric dose-response models. *Manuscr. preparation* (2015).
10. D Galagate, Ph.D. thesis (University of Maryland, College Park) (2016).
11. K Imai, DA Van Dyk, Causal inference with general treatment regimes: Generalizing the propensity score. *J. Am. Stat. Assoc.* **99**, 854–866 (2004).
12. U Shalit, FD Johansson, D Sontag, Estimating individual treatment effect: generalization bounds and algorithms in *International Conference on Machine Learning.* (PMLR), pp. 3076–3085 (2017).
13. C Shi, D Blei, V Veitch, Adapting neural networks for the estimation of treatment effects. *Adv. neural information processing systems* **32** (2019).
14. C Louizos, et al., Causal effect inference with deep latent-variable models. *Adv. neural information processing systems* **30** (2017).
15. J Yoon, J Jordon, M Van Der Schaar, Ganite: Estimation of individualized treatment effects using generative adversarial nets in *International Conference on Learning Representations.* (2018).
16. S Wager, S Athey, Estimation and inference of heterogeneous treatment effects using random forests. *J. Am. Stat. Assoc.* **113**, 1228–1242 (2018).
17. T Ghosh, Y Ma, X de Luna, Sufficient dimension reduction for feasible and robust estimation of average causal effect. *Stat. Sinica* **31**, pp. 821–842 (2021).
